# Exploration of the role and mechanism of Rhizoma Paridis total saponins in osteosarcoma based on SPI1/LCN2-mediated ferroptosis

**DOI:** 10.3389/fonc.2025.1592862

**Published:** 2025-06-25

**Authors:** Ge Yang, Fenghui Li, Xiongke Hu, Miao Li, Yaoxi Liu, Guanghui Zhu, Qian Tan

**Affiliations:** ^1^ Department of Pediatric Orthopedics, Children’s Hospital Affiliated to Xiangya Medical College of Central South University (Hunan Children’s Hospital), Changsha, Hunan, China; ^2^ Department of Nursing, Hunan Provincial Rehabilitation Hospital, Changsha, Hunan, China

**Keywords:** osteosarcoma, Rhizoma Paridis total saponins, ferroptosis, LCN2, SPI1

## Abstract

**Background:**

Osteosarcoma (OS) is a highly aggressive bone malignancy with limited therapeutic options and poor prognosis, particularly in cases of recurrence or metastasis. Recent studies have identified ferroptosis as a promising therapeutic target in OS, with the SPI1/LCN2 regulatory axis emerging as a critical modulator of this process. We hypothesized that Rhizoma Paridis total saponins (RPTS) exert anti-osteosarcoma effects by inducing ferroptosis through inhibition of the SPI1/LCN2 axis.

**Methods:**

*In vitro* assessments using OS cell lines MG-63 and Saos-2 included cell counting kit-8 (CCK-8) assays for viability, colony formation for proliferation, scratch wound healing for migration, and Transwell chambers for invasion. Ferroptosis markers were evaluated using colorimetric Fe^2+^ detection, flow cytometric apoptosis analysis, DCFH-DA probes for reactive oxygen species (ROS), DTNB colorimetry for glutathione (GSH) levels, and western blot (WB) for SLC7A11 and GPX4 expression. A subcutaneous xenograft mouse model received OS cell injections for *in vivo* validation of tumor growth parameters and protein expression. Bioinformatics analysis was conducted to screen OS-specific differentially expressed genes, followed by validation in both *in vivo* and *in vitro* experiments using RT-qPCR and WB.

**Results:**

RPTS significantly inhibited OS cell viability, proliferation, migration, and invasion capacity while promoting Fe^2+^ accumulation and ROS generation. *In vivo*, RPTS treatment significantly inhibited tumor growth *in vivo*. Bioinformatics analysis identified LCN2 as the core ferroptosis mediator regulated by upstream transcription factor SPI1. RPTS downregulated SPI1 and LCN2 expression *in vitro* and *in vivo*. Both LCN2 overexpression and SPI1 activation reversed RPTS-mediated ferroptosis induction. SPI1 overexpression with LCN2 knockdown attenuated the promoting effect of RPTS on ferroptosis.

**Conclusion:**

RPTS triggers ferroptosis-mediated OS suppression through SPI1/LCN2 axis inhibition, providing a novel therapeutic strategy to improve clinical outcomes in OS management.

## 1 Introduction

Osteosarcoma (OS), a highly aggressive malignancy predominantly affecting adolescents, continues to pose significant clinical challenges due to its elevated mortality rate and propensity for recurrence and metastasis despite advancements in multimodal therapies (1, 2). While systemic chemotherapy and surgical interventions have improved survival outcomes, therapeutic efficacy remains suboptimal, partly attributed to the inherent anti-apoptotic properties of tumor cells (3). Consequently, novel strategies to augment programmed cell death mechanisms in OS represent a critical area of investigation.d.

Emerging as a distinct form of regulated cell death, ferroptosis diverges fundamentally from apoptosis in its biochemical execution (4). This iron-dependent process is characterized by the accumulation of lipid peroxides (LPO) and reactive oxygen species (ROS), driven by Fe²^+^/Fe³^+^-mediated Fenton reactions that catalyze hydrogen peroxide (H_2_O_2_) decomposition (5). Two key regulators of ferroptosis are glutathione peroxidase 4 (GPX4) and solute carrier family 7 member 11 (SLC7A11). GPX4 suppresses ferroptosis by reducing LPO, while SLC7A11 promotes cystine uptake, facilitating glutathione synthesis and maintaining cellular redox homeostasis (6). In recent years, ferroptosis has emerged as a promising therapeutic target in OS. OS cells frequently exhibit apoptosis resistance and redox imbalance, suggesting a potential vulnerability to ferroptosis induction (7, 8). Several studies have demonstrated that targeting ferroptosis-related molecules such as GPX4 and SLC7A11 can inhibit OS cell proliferation and metastasis (9, 10). Furthermore, various natural compounds have shown potential as ferroptosis inducers and are being explored as candidate therapeutics in OS (11, 12).

Against this backdrop, Rhizoma Paridis, a traditional Chinese medicinal herb, and its major bioactive components, Rhizoma Paridis total saponins (RPTS), have attracted attention for their potent antitumor activities. Previous studies have shown that RPTS induces apoptosis in colorectal cancer (13), suppresses OS metastasis by downregulating migration-inducing gene 7 (Mig-7) (14), and triggers ferroptosis in breast cancer models (15). However, whether RPTS can exert its antitumor effects in OS via ferroptosis induction remains unexplored. Given the multi-target characteristics and natural origin of RPTS, we hypothesize that RPTS exerts antitumor effects in OS by inducing ferroptosis. Elucidating the underlying molecular mechanisms, particularly those involving iron homeostasis-related pathways, may not only enhance our understanding of ferroptosis-based therapies but also provide a novel and promising direction for natural product-based intervention in osteosarcoma. Therefore, we hypothesize that RPTS exerts antitumor effects in OS by inducing ferroptosis.

Lipocalin 2 (LCN2) is an iron-binding acute-phase protein that plays critical roles in regulating cellular iron homeostasis, inflammatory responses, and tumor progression. In the context of ferroptosis, LCN2 acts as a negative regulator by chelating extracellular iron, thereby limiting intracellular iron accumulation and suppressing lipid peroxidation (16, 17). Elevated LCN2 expression has been associated with ferroptosis resistance, increased tumor aggressiveness, and poor clinical outcomes in various cancers (16–19), suggesting its potential relevance in OS pathophysiology.

SPI1, also known as PU.1, is a member of the ETS transcription factor family originally characterized for its role in hematopoietic differentiation. Recent studies have revealed its oncogenic potential in multiple solid tumors, where it regulates cancer-related processes such as proliferation, survival, and immune evasion (20, 21). In gliomas and esophageal squamous cell carcinoma, SPI1 modulates oncogenic programs through transcriptional control of key pathways (22–25). Notably, SPI1 depletion sensitizes OS cells to cytotoxic insults (26), underscoring its regulatory importance in OS biology. Therefore, investigating whether RPTS induces ferroptosis in OS cells via the SPI1/LCN2 axis could provide valuable mechanistic insights and identify novel therapeutic targets to overcome ferroptosis resistance in osteosarcoma.

To date, there is no research demonstrating the functional crosstalk between RPTS, SPI1, and LCN2 in OS. This study systematically evaluates the ferroptosis-inducing capacity of RPTS in MG-63 and Saos-2 OS cell lines, employing gain-of-function approaches to interrogate SPI1 and LCN2 contributions to ferroptosis regulation. Our findings will provide mechanistic evidence supporting RPTS as a promising phytochemical agent for OS therapy.

## 2 Methods

### 2.1 Bioinformatics analysis

Differentially expressed genes (DEGs) in osteosarcoma (OS) were initially identified from the GSE28424 dataset, which includes transcriptomic profiles of 14 human OS cell lines and 4 normal human bone samples. DEGs were selected based on the criteria of P < 0.05 and |log_2_ fold change| > 1, without multiple testing correction. To delineate ferroptosis-associated molecular drivers in OS pathogenesis, these DEGs were cross-referenced with ferroptosis-related factors obtained from the FerrDb database (http://www.zhounan.org/ferrdb/current/) using the jvenn online tool (https://jvenn.toulouse.inrae.fr/app/example.html) for intersectional visualization. Candidate genes overlapping between these datasets were subsequently subjected to protein-protein interaction (PPI) network analysis via the STRING database (https://string-db.org/), employing a medium-confidence interaction score threshold of 0.4 to identify topologically significant hub proteins. To further elucidate transcriptional regulatory mechanisms, upstream transcription factors governing these core targets were predicted by intersecting the DEGs with transcriptional regulatory data from hTFtarget (https://guolab.wchscu.cn/hTFtarget//#!/), a comprehensive atlas of human transcription factor-gene interactions.

### 2.2 OS cell culture and treatment

The human OS cell lines MG-63 and Saos-2 (Cytion Technologies) were maintained in Dulbecco’s Modified Eagle Medium (DMEM; 11965092, Gibco, Waltham, MA, USA) containing 10% fetal bovine serum (FBS; 26140079, Gibco) and 1% penicillin-streptomycin (15140122, Gibco) at 37°C with 5% CO_2_. Experimental groups, excluding the Control group, were treated with varying concentrations of RPTS (2, 4, and 6 µg/mL). For genetic manipulation studies, subconfluent cells (70-80% confluence) were infected with lentiviral constructs (VectorBuilder) carrying either overexpression vectors (oe-NC, oe-SPI1, oe-LCN2) or knockdown vectors (sh-NC, sh-LCN2 1#, sh-LCN2 2#, sh-LCN2 3#) at a multiplicity of infection (MOI) of 10 for 48 hours. The viral titers used for infection were approximately 1×10^8^ TU/mL. Transduction efficiency was confirmed by fluorescence microscopy or qPCR at 48 hours post-infection. After infection, puromycin (2 μg/mL) was administered for 48 hours to select stably transduced cells and ensure chromosomal integration of the constructs.

### 2.3 CCK-8 assay

The cell counting kit-8 (CCK-8; HY-K0301, MedChemExpress, New Jersey, USA) was applied to measure cell viability. Briefly, cells were planted into 96-well plates (1 × 10^4^ cells/well) and allowed to adhere for 24 hours. Following treatment protocols, culture medium was aspirated, and cells were gently washed with phosphate-buffered saline (PBS). A reaction mixture containing 10 μL CCK-8 reagent and 90 μL serum-free DMEM was added to each well, followed by a 2-hour incubation at 37°C in a humidified atmosphere of 95% air and 5% CO_2_. Absorbance was measured at 450 nm using a microplate reader (BioTek Instruments Inc., Winooski, Vermont, USA) to assess relative vitality.

### 2.4 Colony formation assay

Clonogenic potential was assessed by seeding cells at a density of 2,000 cells per 100-mm culture dish in 10 mL of pre-warmed growth medium at 37°C. Dish were gently rotated to ensure uniform cell distribution prior to incubation in a humidified incubator (5% CO_2_, 37°C) for 2 weeks. Upon macroscopic colony formation, cultures were terminated by supernatant removal, followed by fixation with 5 mL of 4% paraformaldehyde (PFA; 158127, Sigma-Aldrich, St. Louis, MO, USA) for 15 minutes. Fixed colonies were sequentially rinsed with PBS, stained with 10% diluted Giemsa solution (48900, Sigma-Aldrich) for 10 minutes, and air-dried under laminar flow. Quantification was performed by overlaying grid-patterned transparency films on inverted dishes, with colonies containing ≥ 10 cells counted as positive clusters using standardized morphometric criteria.

### 2.5 Scratch assay

The migratory capacity of MG-63 and Saos-2 cells was evaluated using a standardized scratch wound model. Cells were cultured to 100% confluence in dishes to form continuous monolayers. A uniform linear wound was generated in each monolayer using a sterile pipette tip, followed by washes to remove dislodged cellular debris. To minimize proliferation-related confounding effects, wounded monolayers were maintained in serum-free DMEM (A1896702, Thermo Fisher Scientific). Time-lapse imaging was performed at baseline (0 hour) and 24 hours post-wounding using a microscope. Acquired images were subjected to quantitative analysis using ImageJ software (National Institutes of Health, USA), with migration rates calculated.

### 2.6 Transwell assay

To evaluate metastatic potential, OS cells were trypsinized and resuspended in serum-free DMEM at a density of 5 × 10^3^ cells/mL. For migration assays, 200 μL cell suspension (1 × 10^4^ cells) was seeded into the upper chamber of Transwell inserts (without Matrigel coating). Invasion assays utilized Matrigel-precoated inserts, with identical cell loading parameters. The lower chambers received 500 μL DMEM supplemented with 10% FBS (A1896702, Thermo Fisher Scientific) as a chemoattractant. Following 48-hour incubation, non-migratory cells on the upper chamber were removed. Transmigrated cells in the lower chamber were fixed with 4% PFA, stained with crystal violet (198099, Merck, Darmstadt, Germany), and photographed using a Nikon Eclipse E200 microscope (Nikon Corporation, Tokyo, Japan). Quantitative analysis was performed using ImageJ software.

### 2.7 Apoptotic profiling via flow cytometry

Cellular apoptosis was quantified using the ApoDETECT Annexin V-FITC Dual Staining Kit (331200, Thermo Fisher Scientific) following the manufacturer’s instructions. Briefly, cells were harvested by trypsinization, quenched with complete medium, and pelleted by centrifugation at 1,000 rpm for 5 minutes. After discarding the culture medium, cells were resuspended at a density of 1 × 10^6^ cells/mL. Aliquots of 100 µL cell suspension were co-stained with 5 µL Annexin V-FITC and 5 μL propidium iodide (PI) working solution, followed by a 10-minute incubation in the dark at room temperature. Stained cells were immediately analyzed on a CytoFLEX LX flow cytometer (Beckman Coulter).

### 2.8 Intracellular Fe^2+^ quantification

Cellular Fe²^+^ levels were determined using the Iron Colorimetric Assay Kit (EEA008, Thermo Fisher Scientific). Briefly, cells were lysed in RIPA buffer (89900, Thermo Fisher Scientific) on ice for 15 minutes. Lysates were clarified by centrifugation at 15,000 × g for 10 minutes at 4°C, and supernatants were collected. Aliquots were loaded onto 96-well plates, followed by sequential addition of assay reagent as per the manufacturer’s instructions. Afterwards, plates were incubated at 37°C for 30 minutes and absorbance was measured at 593 nm using a microplate reader.

### 2.9 ROS quantification

Cellular ROS generation was assessed using the 2’,7’-dichlorodihydrofluorescein diacetate (DCFH-DA) fluorescent probe (C400, Thermo Fisher Scientific). Harvested cells were trypsinized, pelleted by centrifugation, and resuspended in serum-free medium containing 10 µM pre-diluted DCFH-DA. After 30-minute incubation in the dark at 37°C with intermittent vortexing (5-minute intervals), cells were centrifuged at 1,000 × g and 25°C for 5 minutes. Parallel positive controls were treated with 100 µM tert-butyl hydroperoxide (TBHP). Fluorescence images were obtained using a fluorescence microscope (Olympus) and quantitatively analyzed using ImageJ software, with ROS fluorescence intensity normalized to cell counts (per 10^6^ cells).

### 2.10 Intracellular reduced glutathione quantification

Harvested cells were lysed and mechanically homogenated. The homogenate was centrifuged at 12,000 rpm for 10 minutes at 4°C to pellet cellular debris, and the clarified supernatant was collected. For enzymatic analysis, 100 µL of supernatant was aliquoted, and intracellular GSH levels were measured using the reduced GSH colorimetric assay kit (EIAGSHC, Thermo Fisher Scientific). GSH levels were normalized to total protein concentration (per mg protein) quantified via bicinchoninic acid (BCA) assay.

### 2.11 Reverse transcription-quantitative polymerase chain reaction

Total RNA was isolated using TRIzol reagent (R1030, Prilai, Beijing, China) and treated with RNase-free DNase I (10104159001, Meark KGaA, Darmstadt, Germany) to eliminate genomic DNA contamination. The integrity of RNA samples was spectrophotometrically verified with the HD-UV90 spectrophotometric system (Shandong Hold Electronic Technology Co. Ltd., Weifang, China), strictly adhering to the manufacturer’s operational guidelines. For cDNA synthesis, 2 μg of total RNA underwent RT with the Vazyme DLR102 SynScript^®^ III One-Step RT Kit (DLR102, Vazyme Biotech Co., Ltd., Nanjing, China). RT-qPCR amplification was performed on a thermal cycler (Applied Biosystems, California, USA) in 20 μL reactions, with cycling parameters: initial denaturation at 95°C for 10 minutes, followed by 40 cycles of denaturation at 95°C for 15 seconds, annealing at 60°C for 30 seconds, and extension at 72°C for 30 seconds. Target genes (LCN2 and SPI1) were normalized to the endogenous control GAPDH, and relative mRNA expression levesl were calculated using the 2^-ΔΔCt^ method (27). The specific primer sequences used for RT-qPCR are listed in **Table 1**.

**Table 1:** The primer sequences for RT-qPCR.

Gene	Primer sequences (5′-3′)	
LCN2	Forward	GTGAGCACCAACTACAACCAGC
	Reverse	GTTCCGAAGTCAGCTCCTTGGT
SPI1	Forward	GACACGGATCTATACCAACGCC
	Reverse	CCGTGAAGTTGTTCTCGGCGAA
GAPDH	Forward	GTCTCCTCTGACTTCAACAGCG
	Reverse	ACCACCCTGTTGCTGTAGCCAA

### 2.12 Western blot analysis

Proteins were extracted using RIPA lysis buffer (89900, Thermo Fisher Scientific, Rockford, Illinois, USA) through 30-minute ice incubation with intermittent vortexing at 5-minute intervals. Post-centrifugation of cellular lysates (12,000 rpm, 10 minutes, 4°C), the clarified supernatants were harvested for total protein quantification via BCA protein assay kit (23227, Thermo Fisher Scientific). Protein aliquots (equal loading) were resolved by SDS-PAGE and transferred to PVDF membranes (88518, Thermo Fisher Scientific). Membranes were sequentially blocked with 5% skimmed milk powder for 1 hour, followed by overnight incubation at 4°C with primary antibodies, including anti-SLC7A11 (1:1,000, PA1-16893, Thermo Fisher Scientific), anti-GPX4 (1:10,000, MA5-32827, Thermo Fisher Scientific), anti-LCN2 (1:1,000, PA5-115502, Thermo Fisher Scientific), anti-SPI1 (1:1,000, ab227835, Abcam, Cambridge, Massachusetts, USA), and anti-GAPDH (1:5,000, 4A9L6, Thermo Fisher Scientific). After washes, membranes were probed with horseradish peroxidase (HRP)-conjugated goat anti-rabbit IgG secondary antibody (1:10,000 31460, Thermo Fisher Scientific) at room temperature for 1 hour. Finally, protein bands were visualized using enhanced chemiluminescence (ECL) substrate (32106, Thermo Fisher Scientific) and quantified densitometrically using ImageJ software.

### 2.13 Animal modeling and experimental procedures

All animal experiments were conducted in compliance with protocols approved by the Animal Ethics Committee of Hunan Evidence-based Biotechnology Co., Ltd. (Approval No. ABXZ2312039). Four-week-old male BALB/c nude mice (Fujian Anbuli Biotechnology Co., Ltd., Fuzhou, China) were randomized into six experimental cohorts (n = 6/group): control, RPTS, RPTS + oe-NC, RPTS + oe-SPI1, RPTS + oe-SPI1 + sh-NC, RPTS + oe-SPI1 + sh-LCN2. MG-63 OS cells (1 × 10^6^ cells/mouse) were implanted subcutaneously into the right flank. Except for the control group (administer equal volume of physiological saline solution by gavage), tumor-bearing mice received daily oral gavage of 100 mg/kg RPTS starting 7 days post-inoculation for 14 consecutive days. Humane endpoints were predefined as tumor volume > 2000 mm^3^, > 20% weight loss, or signs of severe morbidity. On day 21, tumor dimensions (major/minor axes) were measured, and tumor volume (V) was calculated following the formula: V = 1/2 × major axis × minor axis^2^. At the end of the experiment, mice were euthanized by intraperitoneal injection of an overdose of sodium pentobarbital (200 mg/kg; P3761, Sigma-Aldrich, St. Louis, MO, USA). The excised tumors were weighed and fixed for subsequent histopathological evaluation.

### 2.14 Hematoxylin-eosin staining

Tumor specimens were fixed in 4% PFA (158127, Sigma-Aldrich) for 24 hours. Following fixation, tissues were sequentially dehydrated, cleared, paraffin-embedded, and sectioned. For staining, sections were deparaffinized in xylene (534056, Sigma-Aldrich) for 5 minutes, and then rehydrated in ethanol (E7023, Sigma-Aldrich). Nuclei were stained with hematoxylin (HHS16, Sigma-Aldrich) for 5 minutes, followed by differentiation in 1% hydrochloric acid-alcohol and blueing in 0.2% ammonia water. Cytoplasmic counterstaining was performed with eosin (HT110132, Sigma-Aldrich) for 1 minute. After final dehydration and clearing, sections were coverslipped using Organo/Limonene Mount™ (O8015, Sigma-Aldrich). Histomorphological evaluation was conducted under a Nikon Eclipse E200 microscope (Nikon Corporation, Tokyo, Japan).

### 2.15 Immunohistochemistry assay

Tumor sections were incubated in citrate buffer (C9999, Sigma-Aldrich) for antigen retrieval. Endogenous peroxidase activity was quenched via 5-minute incubation with 3% hydrogen peroxide (H_2_O_2_). Non-specific binding was blocked for 60 minutes at room temperature using 5% bovine serum albumin (BSA; A9647, Sigma-Aldrich). Sections were subsequently incubated overnight at 4°C with anti-SLC7A11 (1:100, PA1-16893, Thermo Fisher Scientific) and anti-GPX4 antibodies (1:1,000, MA5-32827, Thermo Fisher Scientific). After that, sections were treated with HRP-conjugated goat anti-rabbit secondary antibody (1:200, 31460, Invitrogen) for 30 minutes at room temperature. Immunoreactivity was visualized with chromogenic reagent 3,3’-diaminobenzidine (DAB; SK-4100, Vector Laboratories, Burlingame, USA). Counterstaining was achieved with hematoxylin, followed by dehydration and mounting using Organo/Limonene Mount™. Microscopic observation and imaging were performed, and protein expression levels were quantitatively analyzed using ImageJ software.

### 2.16 Dual-luciferase reporter gene assay

To investigate SPI1-mediated transcriptional regulation of the LCN2 promoter, wild-type (WT) and mutant (MUT) luciferase reporter constructs were engineered. An approximately 2-kb genomic fragment encompassing the LCN2 promoter region (GRCh38 reference sequence chr9:128147453-128149452) was PCR-amplified from human genomic DNA, incorporating multiple predicted SPI1-binding sites (AGGAAGC, AGGAACT, and AGGAAGG as the top three sequences). The amplified products were double-digested with KpnI and XhoI, and then ligated into the upstream region of the multiple cloning site in the pGL3-Basic vector to generate the wild-type reporter vector (pGL3-LCN2-WT). To disrupt SPI1 binding, site-directed mutagenesis was performed on the predicted binding sites using overlapping extension PCR. Specifically, the site at positions 716-722 (reverse strand, sequence AGGAAGC) was mutated to ACCAAGC, the site at 594-600 (reverse strand, AGGAACT) was mutated to ACTAACT, and the sites at 1,374-1,380 or 1,777-1,783 (forward strand, AGGAAGG) were mutated to ATTAAGG. These mutations were designed to disrupt the conserved bases of the typical SPI1 recognition motif “AGGAAG”, aiming to significantly reduce its binding affinity. The mutant promoter fragments, identical in length to the WT, were also double-digested with KpnI and XhoI and cloned into the upstream region of the pGL3-Basic vector’s multiple cloning site, generating the mutant reporter plasmid (pGL3-LCN2-MUT). All constructs were sequence-validated by Sanger sequencing to ensure the accuracy of the inserted sequences and mutation sites. For functional assays, the WT or MUT plasmids were co-transfected with pcDNA3.1(+)-SPI1 overexpression plasmid (or empty vector control) into MG-63 and Saos-2 OS cells using Lipofectamine 2000 (11668-019, Thermo Fisher Scientific, California, USA). Forty-eight hours after transfection, dual-luciferase activity was measured using the Dual-Luciferase Reporter Assay System (E1910, Promega, USA) according to the manufacturer’s instructions. The regulatory effect of SPI1 on the LCN2 promoter was evaluated by comparing luciferase activity.

### 2.17 Chromatin immunoprecipitation-qPCR

ChIP was performed using the Imprint^®^ Ultra ChIP Kit (CHP2NC, Merck, Darmstadt, Germany) according to the manufacturer’s instructions. Briefly, protein-DNA crosslinking was induced by treating cell cultures with PFA (F8775, Merck, Darmstadt, German), followed by quenching with glycine (G7126, Merck, Darmstadt, German). Cells were then mechanically detached, pelleted by centrifugation at 1,000 × g for 5 minutes at 4°C, and resuspended in ChIP lysis buffer (750 μL per 10^7^ cells) for 10-minute ice incubation. Chromatin fragmentation was achieved through optimized ultrasonication to yield DNA fragments. To ensure the specificity of chromatin enrichment and minimize background noise, parallel immunoprecipitation reactions were performed using either an anti-SPI1 antibody (ab227835, Abcam, Cambridge, MA, USA) or a species- and isotype-matched IgG control antibody (SAB5600195, Sigma-Aldrich, St. Louis, MO, USA). Following magnetic bead purification, crosslinks were reversed using proteinase K (20 mg/mL). DNA was purified using the PCR Purification Kit and quantified through qPCR.

### 2.18 Statistical analysis

All quantitative data were expressed as mean ± standard deviation (SD) from biologically independent replicates. Normality distribution was verified employing Shapiro-Wilk testing prior to parametric analysis. Two-group comparisons were analyzed using t-test. Multiple comparisons among three or more sets of data were analyzed through one-way or two-way analysis of variance (ANOVA) as appropriate, followed by Tukey’s *post hoc* testing. Statistical computations were performed in Prism 9 (GraphPad Software, San Diego, CA), with biological and technical replicates specified in respective figure legends. A threshold of *P* < 0.05 established statistical significance for all analyses.

## 3 Results

### 3.1 RPTS exerts multi-faceted anti-tumor effects on OS cells

**Figure 1:**
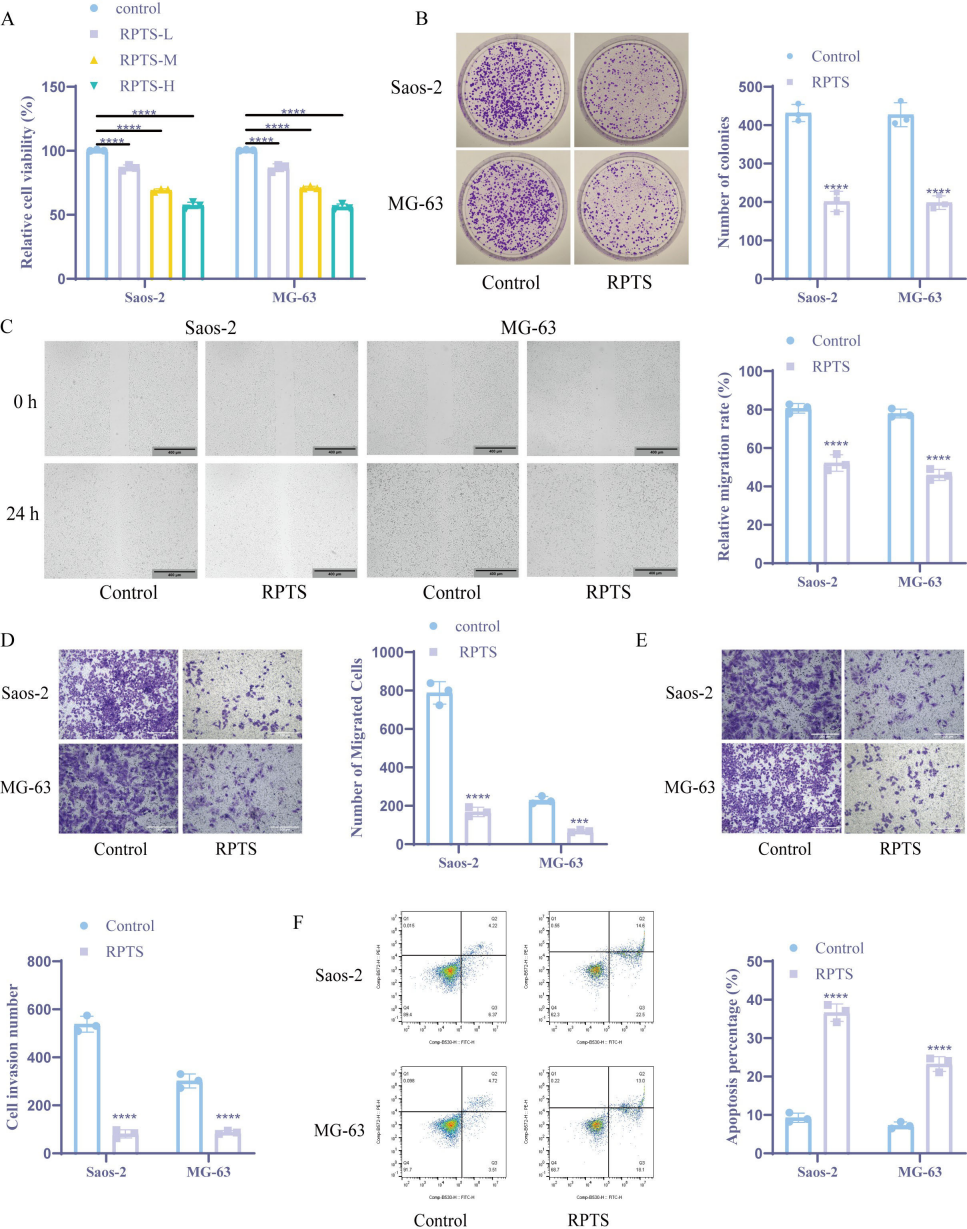
RPTS inhibits OS cell proliferation, migration, and survival. **(A)** Dose-dependent suppression of viability in Saos-2 and MG-63 cells quantified through CCK-8 assay after RPTS exposure (n = 3). **(B)** Colony formation capacity assessed by crystal violet-stained clonogenic survival assay following culture with 6 μg/mL RPTS (n = 3). **(C)** Representative phase-contrast images (100× magnification; scale bar = 400 μm) of scratch wound healing demonstrating impaired migration kinetics post-wounding (n = 3). **(D)** Transwell assay for Saos-2 and MG-63 cell migration (200× magnification; scale bar = 200 μm; n = 3). **(E)** Matrigel invasion potential analyzed through Transwell experiment (200× magnification; scale bar = 200 μm; n = 3). **(F)** Apoptotic induction measured by Annexin V/PI dual staining through flow cytometry (n = 3). Three or more sets of data were analyzed using one-way or two-way ANOVA, followed by Tukey’s *post hoc* testing. ***P < 0.001, ****P < 0.0001.

Dose-response evaluation through CCK-8 viability assays demonstrated concentration-dependent viability inhibition in MG-63 and Saos-2 OS cell lines. Treatment with 6 μg/mL RPTS (designated RPTS-H) reduced cell viability by approximately 43% in Saos-2 cells (from 100.29 ± 0.52 to 57.39 ± 2.48) and by approximately 44% in MG-63 cells (from 100.38 ± 0.38 to 56.15 ± 2.25) compared to untreated controls (**Figure 1A**), thereby establishing this concentration as the working dose for subsequent experiments. Clonogenic survival assays further demonstrated marked anti-proliferative effects, as RPTS treatment decreased colony-forming capacity by approximately 53% in Saos-2 cells (from 431.67 ± 22.37 to 201.33 ± 26.31) and by approximately 54% in MG-63 cells (from 427.33 ± 31.21 to 198.33 ± 17.90) relative to vehicle-treated cells (**Figure 1B**). Cell migration dynamics were interrogated through complementary functional approaches. Scratch wound closure assays showed that RPTS treatment reduced the migration rate of OS cells by approximately 35% in Saos-2 (from 80.63 ± 2.48 to 52.17 ± 4.27) and by approximately 41% in MG-63 (from 77.95 ± 2.25 to 45.98 ± 2.89) (**Figure 1C**). Transwell migration assays revealed a similar decrease in migratory capacity, with a reduction of approximately 79% in Saos-2 (from 788.00 ± 58.59 to 168.33 ± 24.58) and 70% in MG-63 (from 229.33 ± 19.55 to 68.33 ± 6.51) (**Figure 1D**). Matrigel invasion assays demonstrated more pronounced anti-metastatic effects, with RPTS treatment decreasing infiltrative capacity by approximately 71% in Saos-2 (from 301.67 ± 29.02 to 87.67 ± 7.02) and by 85% in MG-63 (from 538.00 ± 33.06 to 81.33 ± 17.16) (**Figure 1E**). Apoptotic induction was confirmed through flow cytometry analysis, revealing an increase in apoptotic rate by approximately 296% in Saos-2 (from 9.26 ± 1.21 to 36.63 ± 2.28) and 219% in MG-63 (from 7.29 ± 0.88 to 23.28 ± 1.91) following RPTS exposure (**Figure 1F**). Collectively, these findings establish RPTS as a multi-mechanistic inhibitor of OS cell proliferation, motility, and survival.

### 3.2 RPTS induces ferroptosis in both *in vivo* and *in vitro* OS models

**Figure 2:**
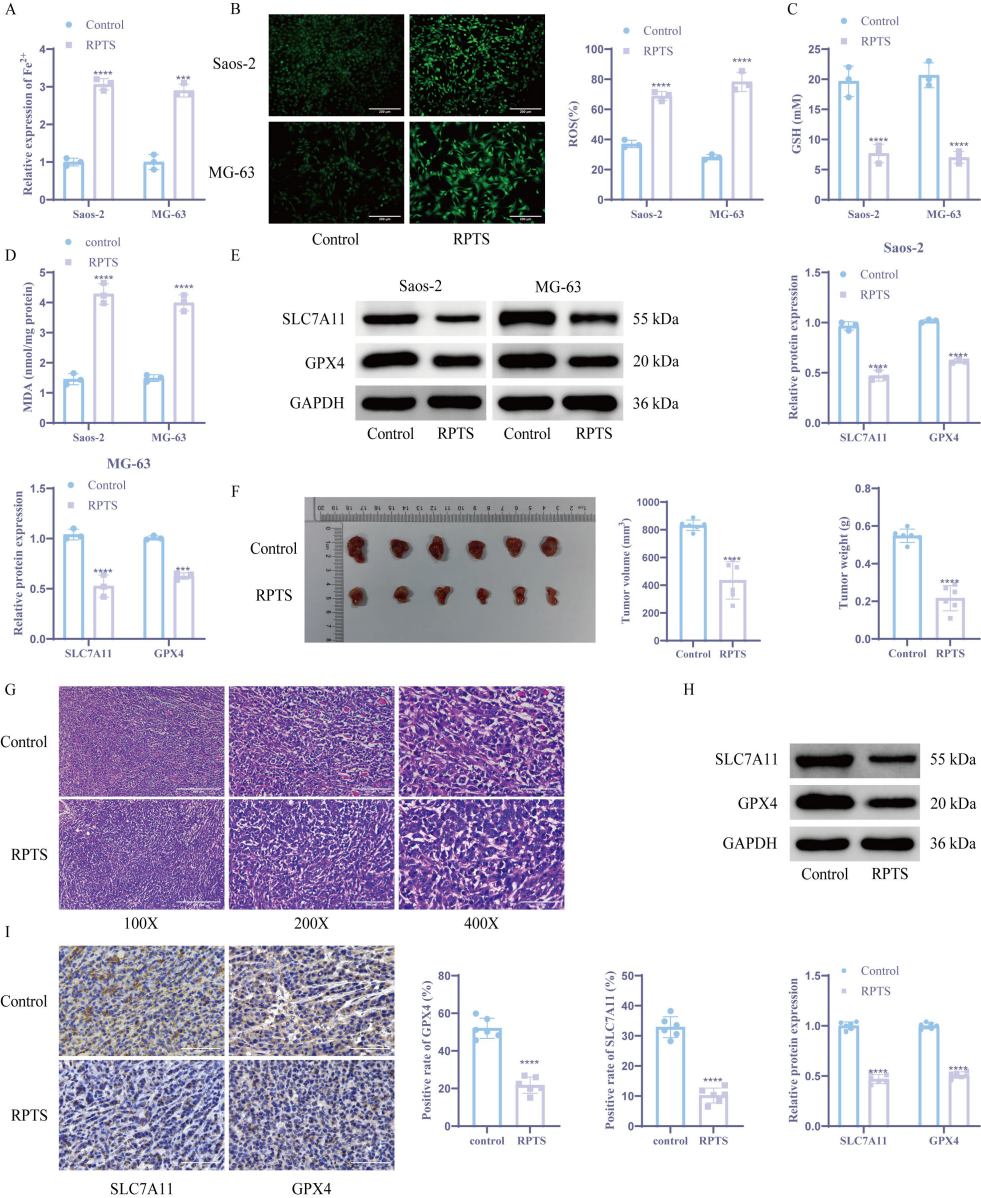
RPTS induces ferroptosis in both *in vivo* and *in vitro* OS models. **(A)** The colorimetric method was used to detect Fe^2+^ levels (n = 3). **(B)** DCFH-DA probe method for detecting ROS (n = 3). The magnification of the image is 200 times (scale bar = 200 μm). **(C)** DTNB detects GSH levels (n = 3). **(D)** Detection of MDA levels in tumor cells (n = 3). **(E)** WB detection of SLC7A11 and GPX4 expression (n = 3). **(F)** Tumor volume and weight detection (n = 6). **(G)** HE staining for detecting histological changes in tumor tissue (n = 6). The image magnification is 100X, 200X, 400X (scale bar 400 μm, 200 μm, 100 μm) **(H-I)** WB and IHC detection of SLC7A11 and GPX4 expression. (n = 6). The magnification of the image is 400 times (scale bar = 100 μm). The detection between the two groups was analyzed using t-test. Three or more sets of data were analyzed using one-way ANOVA and subjected to *post hoc* testing using Tukey’s. ***P < 0.001, ****P < 0.0001.

Ferroptotic modulation by RPTS was investigated through multi-platform biochemical assays and xenograft validation. Intracellular Fe²^+^ quantification using the colorimetric method showed that the intracellular Fe^2+^ level significantly increased after RPTS intervention (Saos-2, Control: 1.00 ± 0.10 to RPTS: 3.07 ± 0.15; MG-63, Control: 1.00 ± 0.20 to RPTS-H: 2.90 ± 0.17), representing a 3.07-fold and 2.90-fold increase, respectively (**Figure 2A**). ROS accumulation was confirmed via the DCFH-DA probe method, demonstrating that the intracellular ROS concentration increased significantly following RPTS intervention (Saos-2, Control: 36.99 ± 2.47 to RPTS: 68.87 ± 3.10, a 1.86-fold increase; MG-63, Control: 28.26 ± 1.81 to RPTS-H: 78.27 ± 6.39, a 2.77-fold increase) (**Figure 2B**). Quantification of reduced GSH using DTNB revealed that intracellular GSH levels markedly decreased following RPTS exposure, with a reduction of 61.0% in Saos-2 cells (Control: 19.67 ± 2.52 to RPTS: 7.67 ± 1.53) and 66.1% in MG-63 cells (Control: 20.67 ± 2.08 to RPTS-H: 7.00 ± 1.00) (**Figure 2C**). Lipid peroxidation was evaluated by measuring MDA levels, which exhibited a 2.96-fold increase in Saos-2 cells (Control: 1.45 ± 0.19 to RPTS: 4.29 ± 0.34) and a 2.68-fold increase in MG-63 cells (Control: 1.49 ± 0.11 to RPTS-H: 3.99 ± 0.27) after RPTS intervention (**Figure 2D**). WB analysis of ferroptosis regulators revealed marked downregulation of GPX4 and SLC7A11 following RPTS therapy (**Figure 2E**). For *in vivo* validation, a mouse xenograft tumor model was established by subcutaneous injection of MG-63 cells into the right flanks of mice. RPTS treatment resulted in a 47.8% reduction in tumor volume (Control: 832.42 ± 37.75 to RPTS: 434.80 ± 134.84, 95% confidence interval -525.0 to -270.3) and a 60.0% reduction in tumor weight (Control: 0.55 ± 0.04 to RPTS: 0.22 ± 0.07, 95% confidence interval -0.4005 to -0.2629) compared to the control group (**Figure 2F**). Histopathological assessment via HE staining demonstrated decreased cellular atypia, reduced nuclear/cytoplasmic ratios, and diminished mitotic figures with concomitant stromal regression after RPTS treatment, indicating a decrease in proliferation activity and a reduction in malignancy (**Figure 2G**). WB and IHC analyses confirmed that RPTS downregulated SLC7A11 and GPX4 expression in excised tumors (**Figures 2H, I**). These results indicate that RPTS can promote ferroptosis in both *in vivo* and *in vitro* OS models.

### 3.3 LCN2 mediates RPTS-induced ferroptosis in OS

**Figure 3:**
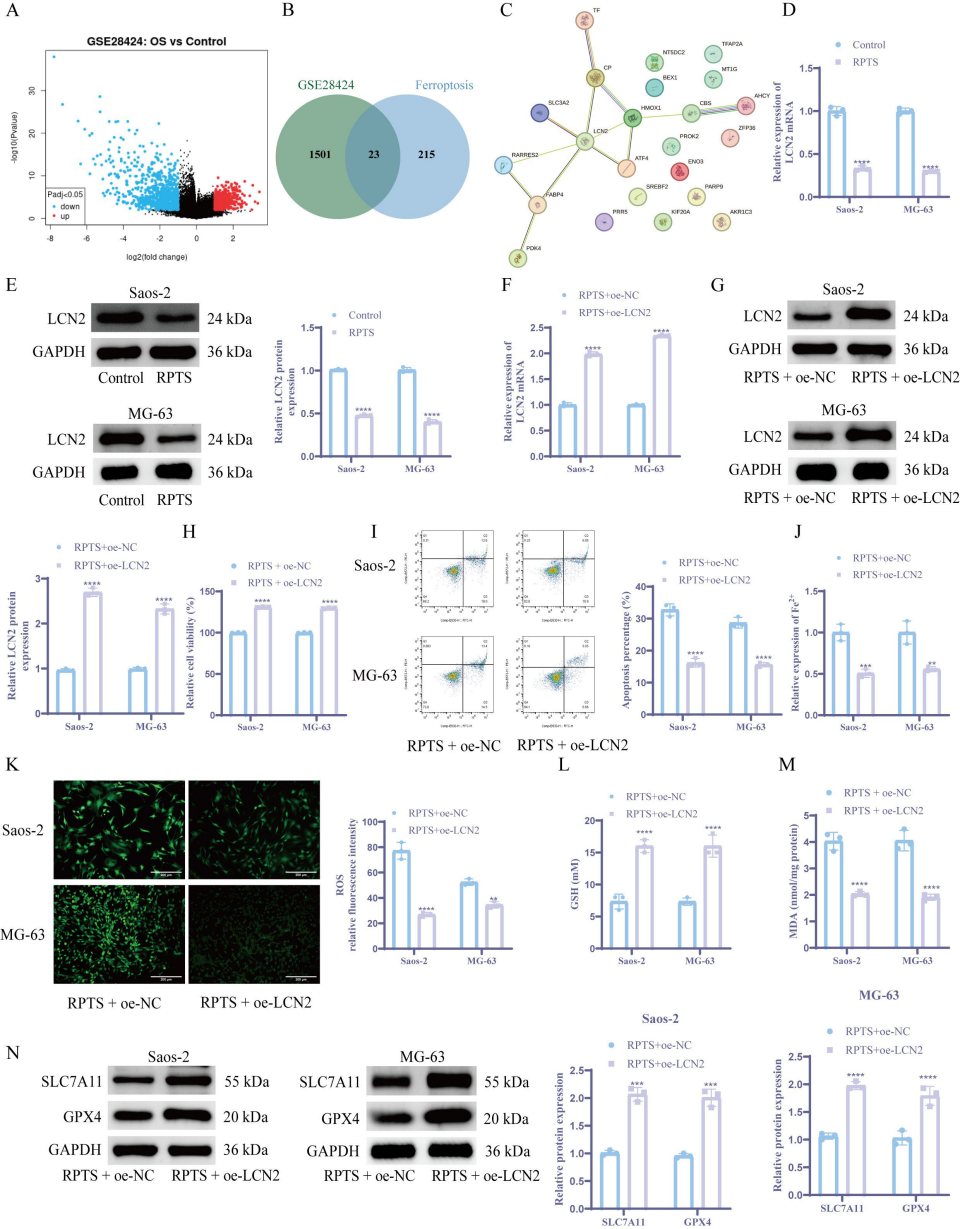
LCN2 modulates RPTS-driven ferroptosis in OS. **(A)** Volcano plot of DEGs from GSE28424. **(B)** Venn plot of the intersection between ferroptosis-related factors and OS-specific DEGs downloaded from the FerrDb database. **(C)** STRING-derived PPI network with LCN2 as topological hub. **(D, E)** LCN2 suppression post-RPTS via RT-qPCR **(D)** and WB **(E)** (n = 3). **(F, G)** Successful LCN2 overexpression validation via RT-qPCR **(F)** and WB **(G)** (n = 3). **(H)** CCK-8 assay for viability in LCN2-overexpressing cells under RPTS treatment (n = 3). **(I)** Apoptosis attenuation in LCN2-overexpressing populations by flow cytometry (n = 3). **(J)** Fe²^+^ quantification via the colorimetric method. **(K)** ROS visualization using DCFH-DA (200× magnification; scale bar = 200 μm; n = 3). **(L)** GSH restoration in LCN2-overexpressing cells based on DTNB (n = 3). **(M)** Detection of MDA levels in LCN2-overexpressing cells (n = 3). **(N)** WB detection of SLC7A11 and GPX4 expression in LCN2-overexpressing cells (n = 3). Three or more sets of data were analyzed using one-way ANOVA with Tukey’s *post hoc* testing. **P < 0.01, ***P < 0.001, ****P < 0.0001.

OS-specific DEGs were identified through bioinformatics analysis of GSE28424 transcriptomic data (**Figure 3A**). Intersection with ferroptosis-related factors from the FerrDb database yielded 23 candidate regulators (**Figure 3B**). PPI network analysis via STRING identified LCN2 as the central hub node among ferroptosis modulators (**Figure 3C**). Following RPTS intervention, RT-qPCR and WB detection demonstrated a substantial decrease in LCN2 expression compared to untreated controls (**Figures 3D, E**). To verify the role of LCN2 in OS cells, we overexpressed LCN2 in OS cell. Overexpression of LCN2 demonstrated a considerable increase in LCN2 mRNA and protein expression versus empty vector controls (**Figures 3F, G**). The CCK-8 assay demonstrated that under RPTS treatment, cell viability was significantly increased in LCN2-overexpressing cells compared to control vector cells, with a 31.1% increase in Saos-2 cells (RPTS + oe-NC: 99.77 ± 0.37 to RPTS + oe-LCN2: 130.89 ± 0.35) and a 30.1% increase in MG-63 cells (RPTS + oe-NC: 99.64 ± 0.43 to RPTS + oe-LCN2: 129.65 ± 0.47) (**Figure 3H**). Flow cytometry analysis revealed that under RPTS treatment, LCN2 overexpression significantly reduced the apoptotic rate compared to non-overexpressing controls, with a 50.5% reduction in Saos-2 cells (RPTS + oe-NC: 32.74 ± 1.89 to RPTS + oe-LCN2: 16.20 ± 1.36) and a 45.6% reduction in MG-63 cells (RPTS + oe-NC: 28.70 ± 1.67 to RPTS + oe-LCN2: 15.60 ± 0.70) (**Figure 3I**). In addition, intracellular levels of ferroptosis biomarkers were markedly decreased in LCN2-overexpressing OS cells under RPTS treatment. Specifically, Fe²^+^ levels were reduced by 49.0% in Saos-2 cells (RPTS + oe-NC: 1.00 ± 0.10 to RPTS + oe-LCN2: 0.51 ± 0.05) and 44.0% in MG-63 cells (RPTS + oe-NC: 1.00 ± 0.14 to RPTS + oe-LCN2: 0.56 ± 0.03). Similarly, ROS levels decreased by 65.1% in Saos-2 cells (77.20 ± 6.61 to 26.91 ± 1.72) and 34.1% in MG-63 cells (52.47 ± 2.56 to 34.57 ± 2.63) (**Figures 3J, K**). GSH homeostasis shifted toward a more reductive state, with GSH levels increased by 118.3% in Saos-2 cells (7.33 ± 1.15 to 16.00 ± 1.00) and 118.3% in MG-63 cells (7.33 ± 0.58 to 16.00 ± 1.73), as determined by the DTNB assay. Meanwhile, lipid peroxidation levels were significantly reduced, as indicated by MDA measurements showing a 49.6% decrease in Saos-2 cells (4.03 ± 0.33 to 2.03 ± 0.09) and a 52.8% decrease in MG-63 cells (4.05 ± 0.39 to 1.91 ± 0.12) (**Figures 3L, M**). WB analysis confirmed ferroptosis pathway reactivation through SLC7A11 and GPX4 upregulation in LCN2-overexpressing populations following RPTS treatment (**Figure 3N**). These mechanistically complementary findings establish LCN2 as a critical negative regulator of RPTS-induced ferroptosis in OS.

### 3.4 SPI1 transcriptionally upregulates LCN2 expression in OS

**Figure 4:**
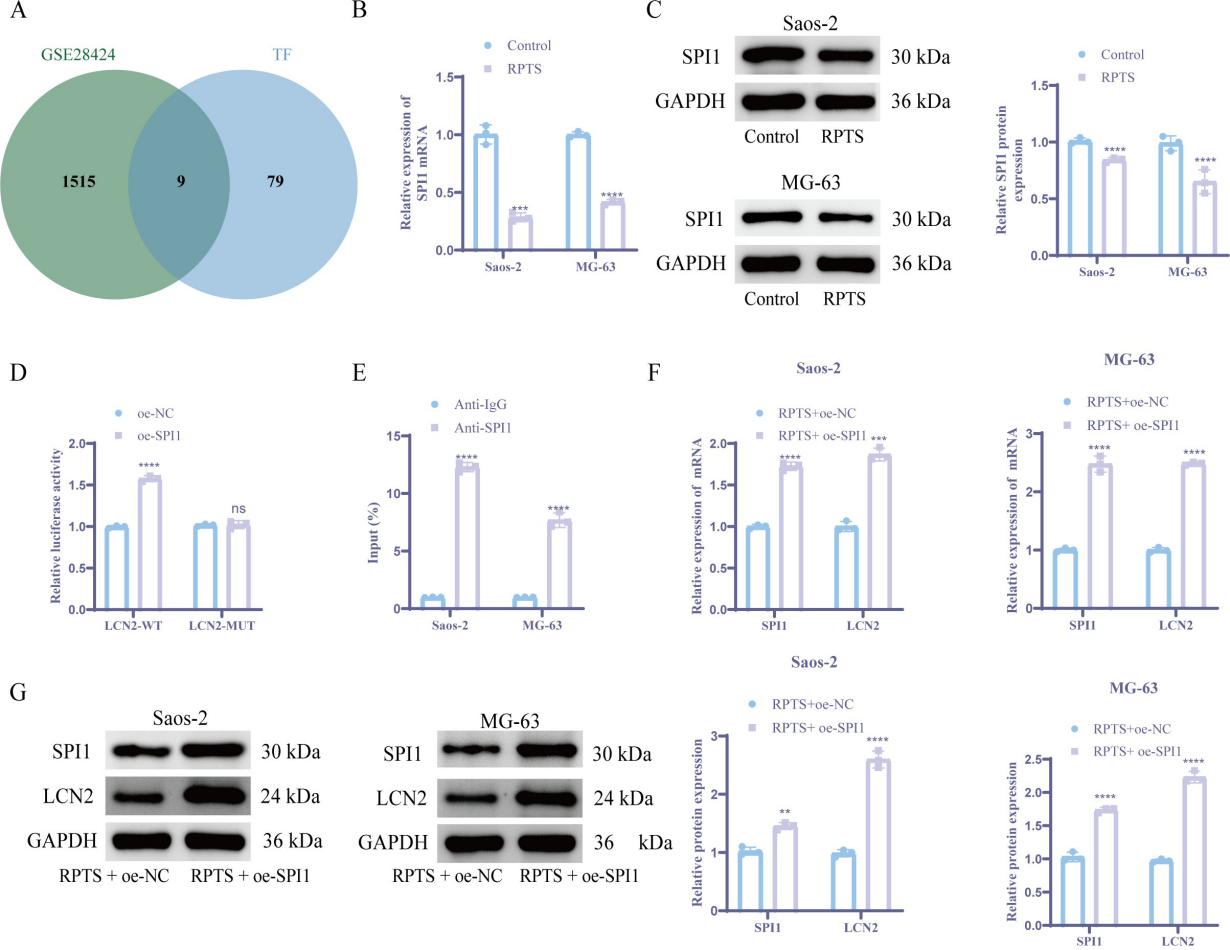
SPI1 acts as an upstream transcription factor of LCN2 and promotes its expression. **(A)** Venn plot of the intersection of LCN2 upstream regulators from hTFtarget and GSE28424 DEGs. **(B, C)** SPI1 downregulation post-RPTS via RT-qPCR **(B)** and WB **(C)** (n = 3). **(D)** Dual-luciferase reporter gene assay comparing WT vs. MUT LCN2 promoter activity (n = 3). **(E)** ChIP-qPCR demonstrating SPI1 binding to LCN2 promoter **(F, G)** Coordinated SPI1/LCN2 upregulation following SPI1 overexpression via RT-qPCR **(F)** and WB **(G)** (n = 3). Three or more sets of data were analyzed using one-way ANOVA with Tukey’s *post hoc* testing. ns *P* > 0.05, **P < 0.01, ***P < 0.001, ****P < 0.0001.

Integrated analysis of DEGs in the GSE28424 dataset and the upstream transcription factors of LCN2 from hTFtarget database identified nine candidate transcription factors. SPI1 emerged as the prioritized target based on its lowest *P* value and maximal survival significance (**Figure 4A**). RT-qPCR and WB detection showed that the expression level of SPI1 was significantly reduced after RPTS intervention versus untreated controls (**Figures 4B, C**). Functional validation through dual-luciferase reporter gene assays demonstrated SPI1-mediated transcriptional activation of the LCN2 promoter. In OS cells, co-transfection of SPI1 with LCN2-WT significantly increased luciferase activity compared to negative controls, while LCN2-MUT showed no significant response (**Figure 4D**). ChIP-qPCR confirmed direct SPI1 binding to the promoter region of LCN2 (**Figure 4E**). To verify the role of SPI1 in OS cells, we performed SPI1 overexpression experiments. RT-qPCR and WB analyses confirmed a significant increase in SPI1 expression following SPI1 overexpression. Notably, overexpression of SPI1 also led to a marked upregulation of LCN2 expression at both mRNA and protein levels (**Figures 4F, G**). This regulatory relationship establishes SPI1, as a direct transcriptional activator of LCN2 in OS pathogenesis.

### 3.5 RPTS inhibits malignant behaviors of OS cells through SPI1/LCN2

**Figure 5:**
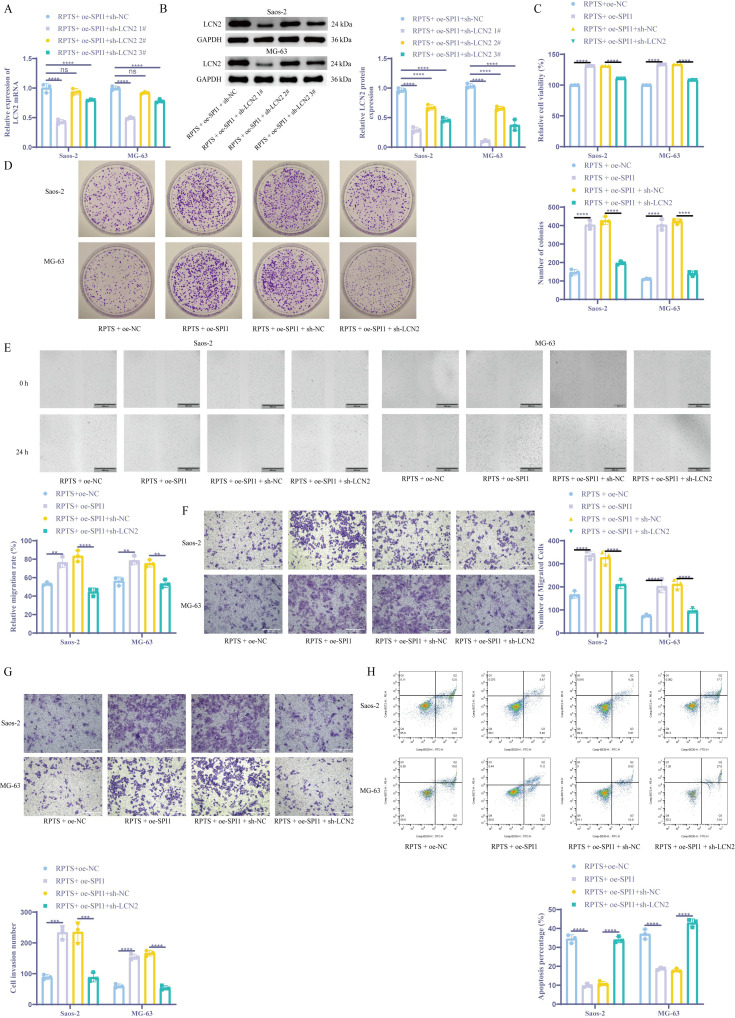
RPTS inhibits malignant behaviors of OS cells through SPI1/LCN2. **(A, B)** LCN2 suppression in combinatorial RPTS + OE-SPI1 + sh-LCN2 #1 treatment via RT-qPCR **(A)** and WB **(B)** (n = 3). **(C)** Viability modulation across treatment groups by CCK-8 assay (n = 3). **(D)** Clonogenic survival under genetic perturbations (n = 3). **(E)** Scratch wound healing progression (100× magnification; scale bar = 400 μm; n = 3). **(F)** Transwell assay for Saos-2 and MG-63 cell migration (200× magnification; scale bar = 200 μm; n = 3). **(G)** Transwell experiment detecting cell invasion (200× magnification; scale bar = 200 μm; n = 3). **(H)** Flow cytometry for cell apoptosis (n = 3). Three or more sets of data were analyzed using two-way ANOVA with Tukey’s *post hoc* testing. ns *P* > 0.05, *P < 0.01, ***P < 0.001, ****P < 0.0001.

Combinatorial genetic manipulation through SPI1 overexpression and LCN2 knockdown in OS cells revealed hierarchical regulatory dynamics. Co-treatment with RPTS + oe-SPI1 + sh-LCN2 1# achieved maximal LCN2 suppression as validated by RT-qPCR and WB (**Figures 5A, B**), thus selecting sh-LCN2 1# for subsequent experiments. Functional characterization via CCK-8 assays demonstrated that, compared with the RPTS + oe-NC group, the RPTS + oe-SPI1 group exhibited a 31.2% increase in cell viability in Saos-2 cells (99.85 ± 0.29 to 131.03 ± 0.15) and a 33.4% increase in MG-63 cells (99.97 ± 0.04 to 133.32 ± 0.28). Conversely, LCN2 knockdown in the context of SPI1 overexpression led to a 15.5% decrease in Saos-2 cells (131.01 ± 0.22 to 110.77 ± 0.06) and an 18.9% decrease in MG-63 cells (133.69 ± 0.19 to 108.28 ± 0.38) (**Figure 5C**). Clonogenic assays revealed that, compared with cells not overexpressing SPI1, cell proliferation ability increased by 2.74-fold in Saos-2 cells (RPTS + oe-NC: 147.33 ± 15.50 to 403.67 ± 22.55) and by 3.63-fold in MG-63 cells (RPTS + oe-NC: 111.00 ± 3.61 to 403.67 ± 29.28) in SPI1-overexpressing cells. Conversely, knocking down LCN2 in the context of SPI1 overexpression resulted in a 53.8% decrease in Saos-2 cells (427.33 ± 20.84 to 197.33 ± 9.07) and a 66.7% decrease in MG-63 cells (422.67 ± 13.65 to 141.00 ± 13.00) (**Figure 5D**). Scratch wound healing and Transwell migration assaysdemonstrated that LCN2 knockdown significantly attenuated the SPI1 overexpression-induced enhancement of cell migration ability. In the scratch assay, SPI1 overexpression increased wound closure by 43.7% in Saos-2 cells (RPTS + oe-NC: 53.25 ± 1.74 to 76.53 ± 5.68) and by 39.6% in MG-63 cells (56.30 ± 4.50 to 78.62 ± 4.69), whereas subsequent LCN2 knockdown reduced wound closure by 46.5% in Saos-2 (RPTS + oe-SPI1 + sh-NC: 83.45 ± 6.02 to 44.65 ± 4.24) and 29.1% in MG-63 cells (75.36 ± 3.86 to 53.43 ± 4.34) (**Figure 5E**). In the Transwell migration assay, SPI1 overexpression led to a 102.5% increase in migrated cell number in Saos-2 cells (RPTS + oe-NC: 166.00 ± 15.13 to 336.33 ± 16.56) and a 170.2% increase in MG-63 cells (75.00 ± 5.57 to 202.67 ± 28.54). LCN2 knockdown reversed this effect, decreasing migration by 35.6% in Saos-2 (327.00 ± 28.35 to 210.67 ± 18.45) and 54.5% in MG-63 cells (211.67 ± 20.84 to 96.33 ± 10.21) (**Figure 5F**). Transwell invasion assays showed that SPI1 overexpression significantly enhanced the invasive ability of OS cells, with a 2.57-fold increase in Saos-2 cells (RPTS + oe-NC: 60.00 ± 6.56 to 154.33 ± 9.07) and a 2.63-fold increase in MG-63 cells (89.00 ± 8.00 to 233.67 ± 21.50). However, knockdown of LCN2 reversed this effect, reducing invasion by 67.6% in Saos-2 cells (RPTS + oe-SPI1 + sh-NC: 166.67 ± 9.07 to 54.00 ± 7.21) and by 62.4% in MG-63 cells (235.00 ± 33.72 to 88.33 ± 14.98) (**Figure 5G**). Flow cytometry analysis demonstrated that SPI1 overexpression markedly suppressed RPTS-induced apoptosis, decreasing the apoptotic rate by 71.6% in Saos-2 cells (RPTS + oe-NC: 34.54 ± 2.16 to 9.81 ± 0.95) and by 49.5% in MG-63 cells (37.04 ± 2.61 to 18.70 ± 0.62). However, simultaneous knockdown of LCN2 reversed this effect, restoring apoptosis to levels comparable to or exceeding the control group. Specifically, apoptosis increased by 211.8% in Saos-2 cells (RPTS + oe-SPI1 + sh-NC: 10.93 ± 1.16 to 34.08 ± 1.65) and by 139.9% in MG-63 cells (17.91 ± 0.89 to 42.97 ± 2.08) (**Figure 5H**). These functional epistasis analyses indicate that RPTS inhibits the malignant behaviors of OS cells through SPI1/LCN2.

### 3.6 RPTS promotes ferroptosis through SPI1/LCN2

**Figure 6:**
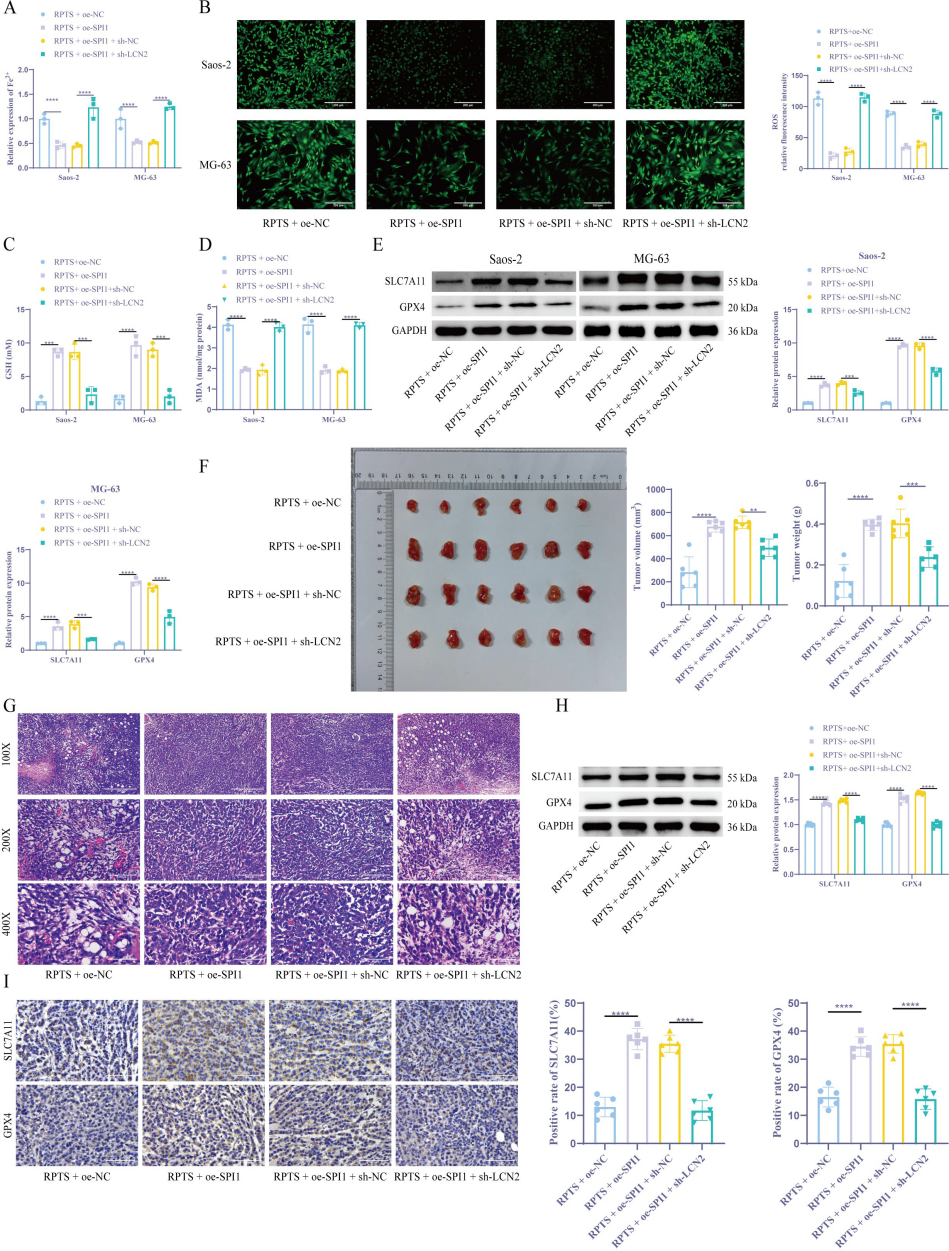
RPTS promotes ferroptosis through SPI1/LCN2. **(A)** Fe²^+^ quantification via the colorimetric method (n = 3). **(B)** ROS visualization by flow cytometry (200× magnification; scale bar = 200 μm; n = 3). **(C)** GSH restoration through DTNB assay (n = 3). **(D)** Detection of MDA levels in OS cells (n = 3). **(E)** WB detection of SLC7A11 and GPX4 expression in OS cells (n = 3). **(F)** Xenograft tumor volume and weight quantification (n = 6). **(G)** HE staining for detecting histopathological changes in tumor tissues (100×, 200×, 400× magnification; scale bar = 400 μm, 200 μm, 100 μm; n = 6). **(H, I)** WB and IHC detection of SLC7A11 and GPX4 expression in xenograft tumors (400× magnification; scale bar = 100 μm; n = 6). Three or more sets of data were analyzed using one-way or two-way ANOVA with Tukey’s *post hoc* testing. *P < 0.01, ***P < 0.001, ****P < 0.0001.

Colorimetric quantification revealed that SPI1 overexpression significantly decreased intracellular Fe²^+^ levels by 54.0% in Saos-2 cells (from 1.00 ± 0.10 to 0.46 ± 0.07) and by 47.0% in MG-63 cells (from 1.00 ± 0.20 to 0.53 ± 0.03). Concurrently, flow cytometry analysis showed that ROS levels dropped by 81.6% in Saos-2 cells (from 113.35 ± 10.05 to 20.81 ± 4.68) and by 60.7% in MG-63 cells (from 88.92 ± 3.76 to 34.91 ± 3.09) following SPI1 overexpression. However, knockdown of LCN2 in the context of SPI1 overexpression led to marked increases in ferroptosis indicators. Specifically, Fe²^+^ levels rose by 173.3% in Saos-2 cells (0.45 ± 0.04 to 1.23 ± 0.21) and by 140.4% in MG-63 cells (0.52 ± 0.03 to 1.25 ± 0.06), while ROS levels surged by 314.7% in Saos-2 (27.73 ± 5.21 to 114.99 ± 6.39) and 125.3% in MG-63 (39.17 ± 4.17 to 88.22 ± 5.90) (**Figures 6A, B**). The DTNB assay demonstrated that GSH levels in cells overexpressing SPI1 increased by approximately fourfold, while shLCN2 #1 co-treatment led to a significant decrease in intracellular GSH levels (**Figure 6C**). Lipid peroxidation mirrored this pattern, as the downregulation of LCN2 reversed the promoting effect of SPI1 overexpression on MDA (**Figure 6D**). Molecular profiling through WB detection confirmed that compared with the RPTS + oe-NC group, the expression levels of SLC7A11 and GPX4 in the RPTS + oe-SPI1 group were significantly increased. However, overexpression of SPI1 while silencing LCN2 resulted in a significant decrease in GPX4/SLC7A11 expression (**Figure 6E**). *In vivo* validation showed that, under RPTS treatment, mice bearing SPI1-overexpressing OS cells exhibited markedly enhanced tumor growth compared to those injected with non-SPI1-overexpressing cells. Specifically, tumor volume increased by 139.9% (from 283.04 ± 133.16 to 679.01 ± 47.52 mm³, 95.00% CI of diff -532.7 to -259.2) and tumor weight increased by 233.3% (from 0.12 ± 0.08 to 0.40 ± 0.03 g, 95.00% CI of diff -0.3728 to -0.1772). However, silencing LCN2 in the context of SPI1 overexpression significantly reversed this effect. Compared with the RPTS + oe-SPI1 + sh-NC group, tumor volume and weight in the RPTS + oe-SPI1 + sh-LCN2 group were reduced by 30.7% (from 715.16 ± 54.78 to 495.31 ± 75.21 mm³, 95.00% CI of diff 83.09 to 356.6) and 40.0% (from 0.40 ± 0.07 to 0.24 ± 0.05 g, 95.00% CI of diff 0.06722 to 0.2628), respectively (**Figure 6F**). HE staining results showed that SPI1 overexpression enhanced cellular pleomorphism, nuclear/cytoplasmic ratios, mitotic figures, and surrounding stroma in tumor tissues, indicating elevated proliferative activity and malignancy. Knockdown of LCN2 reversed these pathological changes (**Figure 6G**). WB and IHC confirmed that under RPTS intervention, compared with mice inoculated with non-SPI1-overexpressing OS cells, mice inoculated with SPI1-overexpressing OS cells exhibited significantly increased expression of SLC7A11 and GPX4. However, compared with the RPTS + oe-SPI1 + sh-NC group, the RPTS + oe-SPI1 + sh-LCN2 group showed significantly decreased SLC7A11 and GPX4 expression (**Figures 6H, I**). These results collecitvely indicate that RPTS may promote ferroptosis in OS, at least in part, through the SPI1/LCN2 signaling axis.

## 4 Discussion

OS is a highly aggressive primary bone malignancy, often occurring in adolescents and pediatric populations, and prone to pulmonary metastasis and dismal prognosis (28). Current therapeutic paradigms encompassing surgical resection combined with neoadjuvant/adjuvant chemotherapy (29) have shown limited efficacy, with high recurrence rate (30) and low 5-year survival rates for metastatic cases reported (31). This clinical urgency underscores the critical need for novel therapeutic strategies targeting OS pathogenesis. Our experimental data reveal that RPTS exerts potent anti-neoplastic effects through dose-dependent inhibition of cellular proliferation, migratory capacity, invasive potential, and metabolic viability in OS models, while concomitantly inducing tumor cell death. Among the various forms of programmed cell death implicated in OS, apoptosis, ferroptosis, and autophagy have garnered significant attention for their roles in tumor suppression, modulation of therapeutic sensitivity, and regulation of the tumor microenvironment (9, 32, 33). Understanding the interplay among these pathways is crucial for advancing precision treatment strategies in OS. While RPTS may influence multiple death modalities, our findings highlight ferroptosis as the predominant mechanism. These findings position RPTS as a promising ferroptosis-inducing adjuvant for OS with potential therapeutic translation pending rigorous preclinical validation.

The emerging tumor-suppressive paradigm of ferroptosis, characterized by an iron-dependent programmed cell death pathway mediated by lethal lipid ROS accumulation and mitochondrial cristae loss, has garnered significant oncological interest (34–36). Our mechanistic investigations demonstrated that RPTS administration significantly elevated intracellular Fe^2+^ concentration and ROS generation, while depleting GSH reserves in both *in vitro* and orthotopic murine OS models. Molecular profiling revealed consistent downregulation of key ferroptosis regulators SLC7A11 and GPX4 across experimental conditions. Although these observations highlight ferroptosis activation, contributions from other regulated death pathways, such as apoptosis and necroptosis, cannot be fully excluded and warrant further investigation. This indicates that RPTS exhibits the effect of conserved ferroptotic activation through SLC7A11-GPX4 axis disruption in OS.

To further dissect upstream regulatory signals, we performed integrative bioinformatics analysis of the GSE28424 dataset and identified LCN2, an iron-chelating oncoprotein implicated in ferroptosis resistance (17), as a critical molecular nexus. LCN2 overexpression correlates with tumor progression in multiple malignancies (18) and serves as a cachexia biomarker in advanced lung cancer (16). Notably, our study found that RPTS treatment induced significant LCN2 suppression, while genetic LCN2 overexpression attenuated ferroptosis induction by RPTS through multiple mechanisms: diminished apoptotic rate, attenuated Fe^2+^ accumulation, ROS normalization, GSH restoration, and SLC7A11/GPX4 protein recovery. These findings established LCN2 as a pivotal ferroptosis checkpoint in OS pathogenesis, and prompted us to investigate upstream regulators of LCN2 to better understand the mechanism by which RPTS modulates ferroptosis.

To identify transcriptional drivers of LCN2, we applied a transcriptional regulatory network analysis, which identified SPI1 as the principal upstream regulator of LCN2 expression. SPI1 is a ETS-family transcription factor that can bind to consensus DNA motifs in target gene promoters to orchestrate oncogenic programs (22), with prior evidence linking SPI1 knockdown to OS cell death (26). Our dual-luciferase reporter gene assays and ChIP-qPCR experiments conclusively demonstrated SPI1-mediated transcriptional activation of LCN2. Importantly, SPI1 overexpression rescued LCN2 expression and largely reversed RPTS-induced ferroptosis in OS cells, whereas LCN2 knockdown reinstated RPTS-induced ferroptotic sensitivity, even in the presence of SPI1 overexpression. Although LCN2 has been reported to be involved in iron homeostasis and immune regulation in various types of cancer, its function in osteosarcoma remains insufficiently studied, and its role as a downstream target of SPI1 in the regulation of ferroptosis has not been clearly elucidated. Previous studies on ferroptosis have primarily focused on classical pathways, such as SLC7A11 and GPX4, with relatively limited exploration of upstream transcriptional regulatory networks (9, 10). Therefore, our study expands the current theoretical framework by proposing that SPI1 may act as a key transcription factor that negatively regulates LCN2 expression, thereby indirectly promoting ferroptosis. This provides a novel potential target for the therapeutic modulation of ferroptosis.

Despite strong *in vitro* and orthotopic evidence supporting the role of the RPTS–SPI1–LCN2 axis in ferroptosis induction, several limitations should be acknowledged. First, potential off-target effects of RPTS on other cell death pathways such as apoptosis and necroptosis remain to be fully characterized. Second, systemic factors including whole-body iron metabolism and baseline oxidative stress may confound ferroptosis sensitivity *in vivo*. These parameters should be quantitatively assessed to evaluate the specificity and safety of ferroptosis induction. Third, our study lacked spontaneous metastasis models and survival endpoints, limiting assessment of long-term efficacy and clinical relevance. Finally, translational steps are needed to move this therapeutic approach toward clinical application. Future work should prioritize structure–activity relationship (SAR)-guided RPTS analog development, identification of SPI1-targeted inhibitors, and stratification of patients based on SPI1/LCN2 expression profiles.

**Figure 7:**
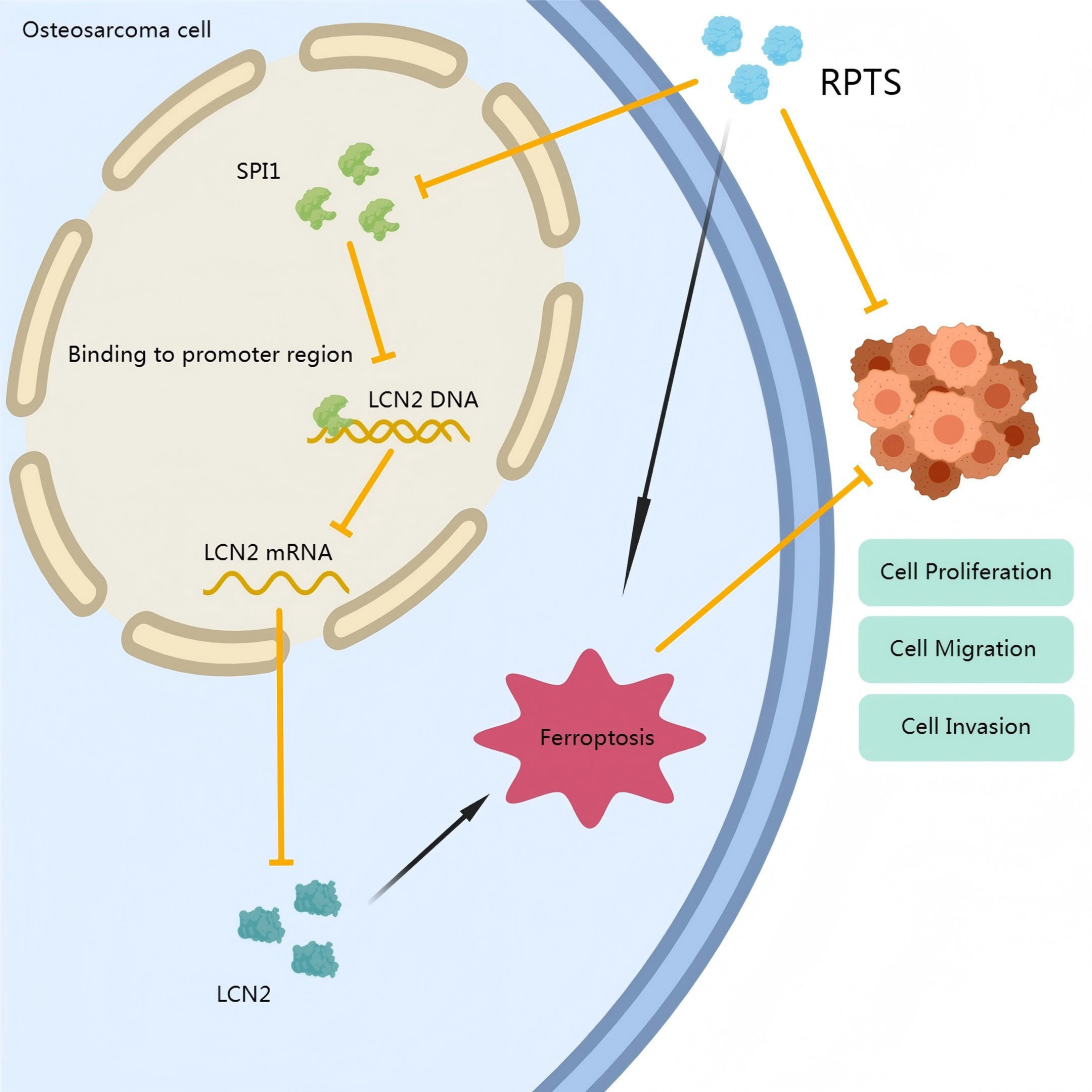
Mechanistic schema of RPTS-induced ferroptosis in OS. RPTS downregulates SPI1 expression, subsequently inhibiting LCN2 transcription. This dual suppression disrupts GSH homeostasis, enhances lipid peroxidation, and depletes Fe²^+^/ROS buffering capacity, ultimately driving iron-dependent programmed cell death through GPX4/SLC7A11 pathway inactivation.

In conclusion, this study establishes a novel ferroptosis-regulating pathway mediated by SPI1-dependent LCN2 transcriptional activation in OS. RPTS-induced ferroptosis, through disruption of this axis, presents a promising therapeutic approach. This proposed mechanism is illustrated in **Figure 7**. These findings not only deepen our understanding of ferroptosis regulation in OS but also provide a mechanistic basis for future ferroptosis-based therapies targeting SPI1 and LCN2.

## 5 Conclusions

Our findings collectively demonstrat that RPTS exerts potent anti-OS effects through SPI1/LCN2 axis inhibition and subsequent ferroptosis induction. The elucidated mechanism involves transcriptional silencing of LCN2 via SPI1 suppression, thereby relieving iron sequestration and permitting lethal lipid peroxidation cascade activation. This study not only proposes a novel ferroptosis-based therapeutic strategy for OS, but also highlights the broader applicability of targeting the SPI1/LCN2 regulatory node in other malignancies characterized by LCN2 overexpression and ferroptosis resistance. Future investigations should prioritize structure–activity relationship (SAR)-guided optimization of RPTS analogs, development of SPI1-selective inhibitors, and biomarker-driven clinical trial strategies to facilitate the translation of these mechanistic insights into precision oncology applications.

## Data Availability

The original contributions presented in the study are included in the article/**Supplementary Material**. Further inquiries can be directed to the corresponding author.
