# Supplementary figures and images for: Exploration of the role and mechanism of Rhizoma Paridis total saponins in osteosarcoma based on SPI1/LCN2-mediated ferroptosis (part 2 of 2)
Source: Front Oncol. 2025 Jun 25;15:1592862. doi: 10.3389/fonc.2025.1592862 (PMC12237685; doi:10.3389/fonc.2025.1592862)

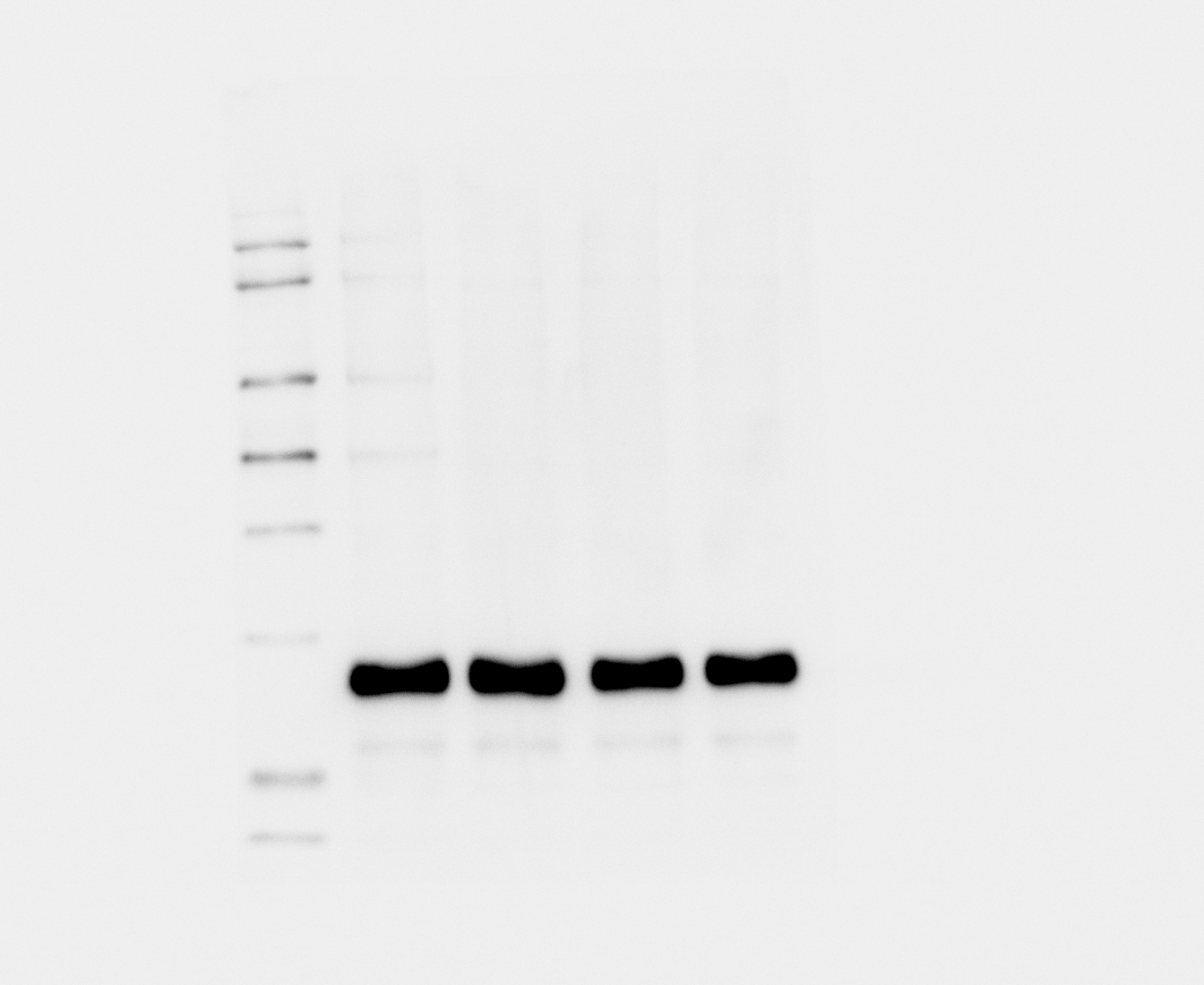

Supplement: Supplementary file 4 [file Supplementaryfile4.zip › Fig 5B/MG-63/GAPDH-3 .tif]

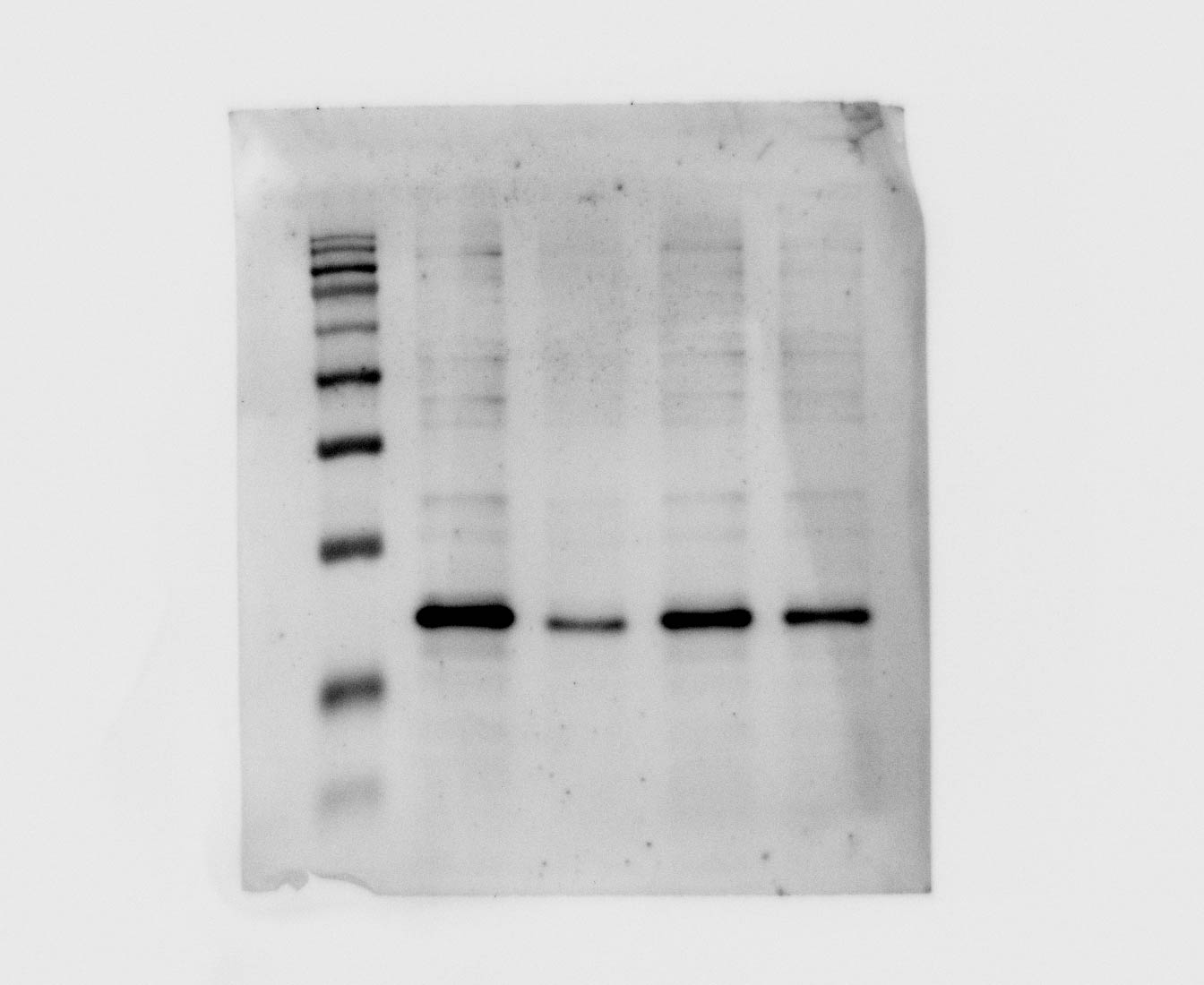

Supplement: Supplementary file 4 [file Supplementaryfile4.zip › Fig 5B/MG-63/LCN2-1 Report.jpg]

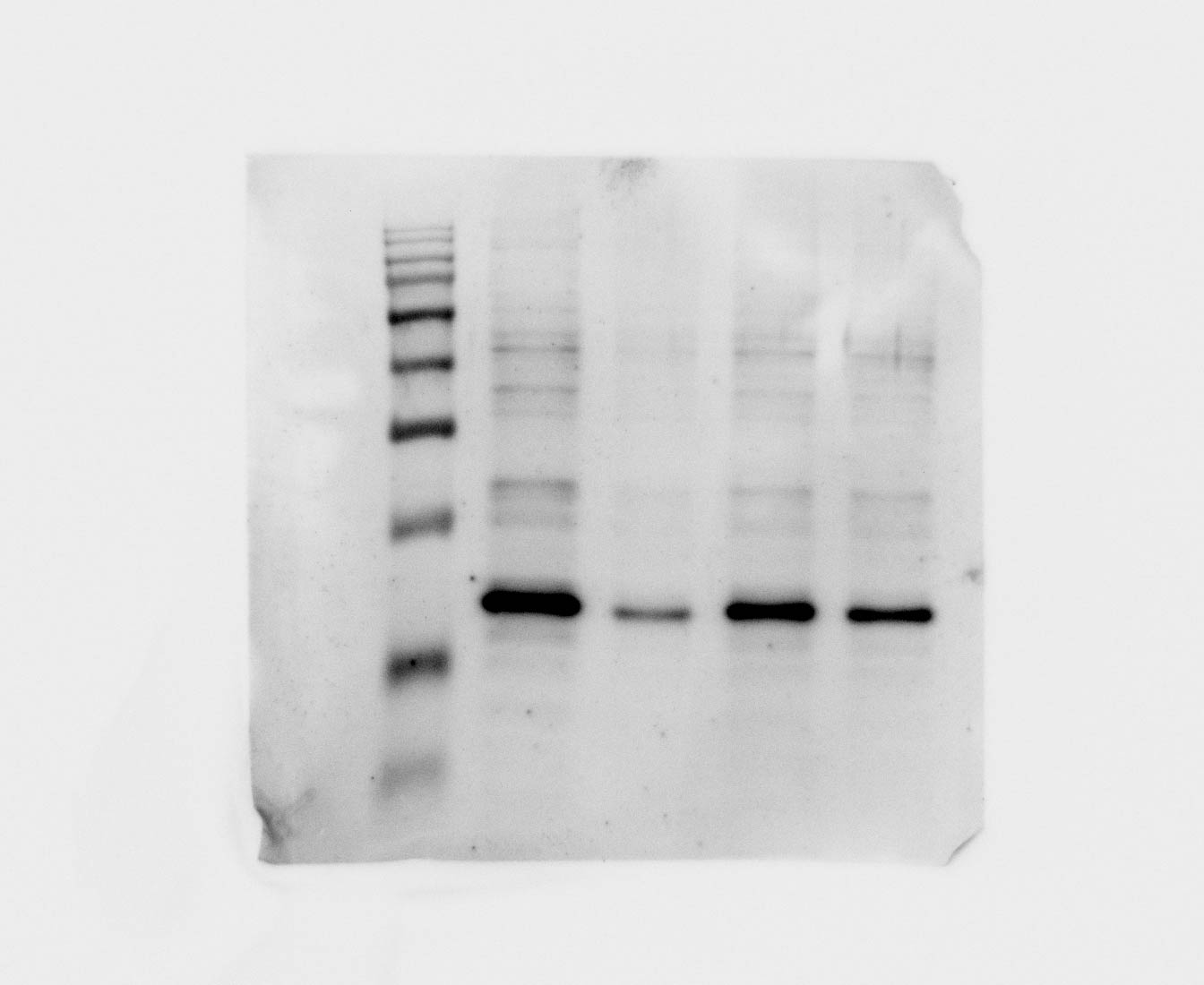

Supplement: Supplementary file 4 [file Supplementaryfile4.zip › Fig 5B/MG-63/LCN2-2.jpg]

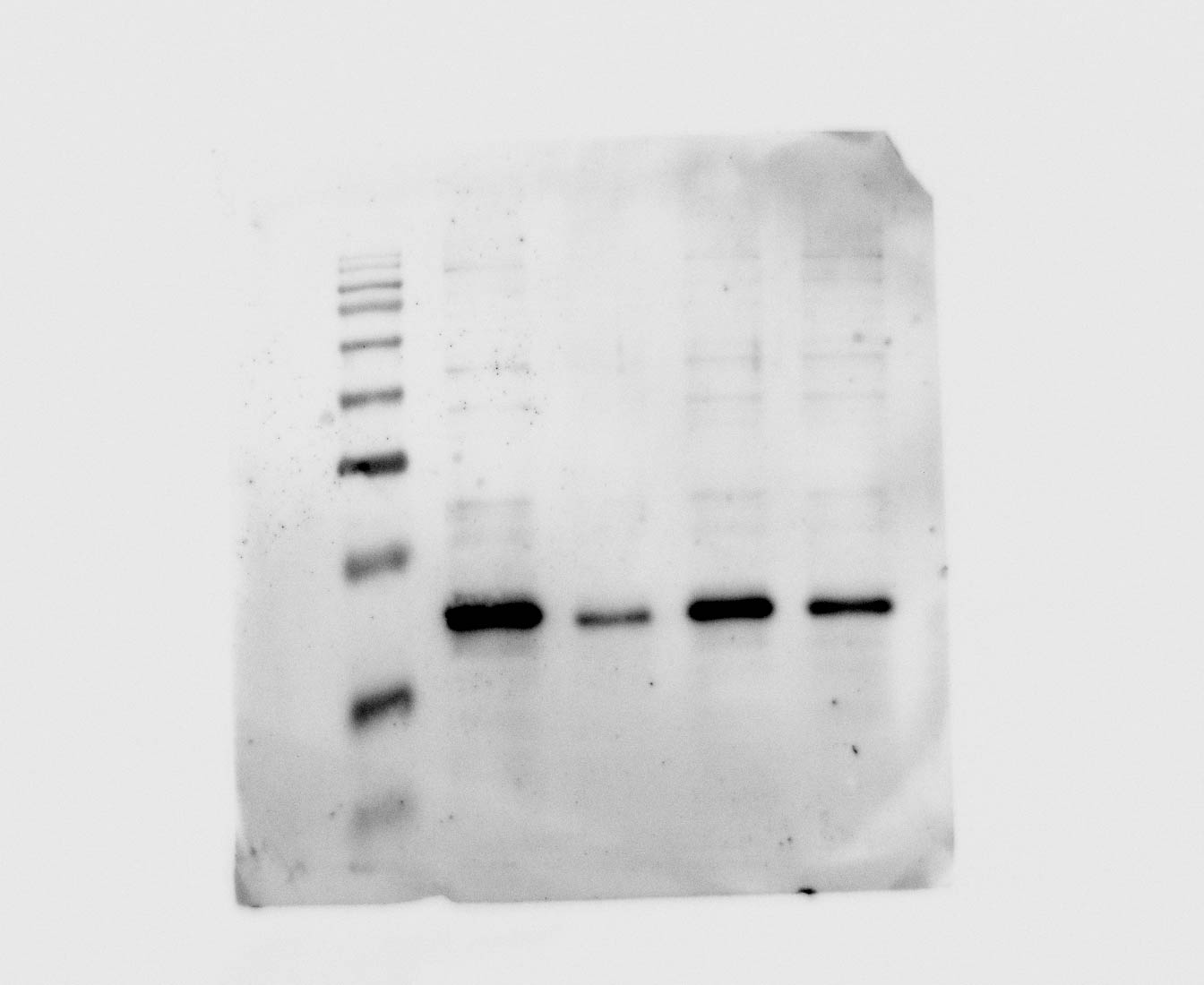

Supplement: Supplementary file 4 [file Supplementaryfile4.zip › Fig 5B/MG-63/LCN2-3.jpg]

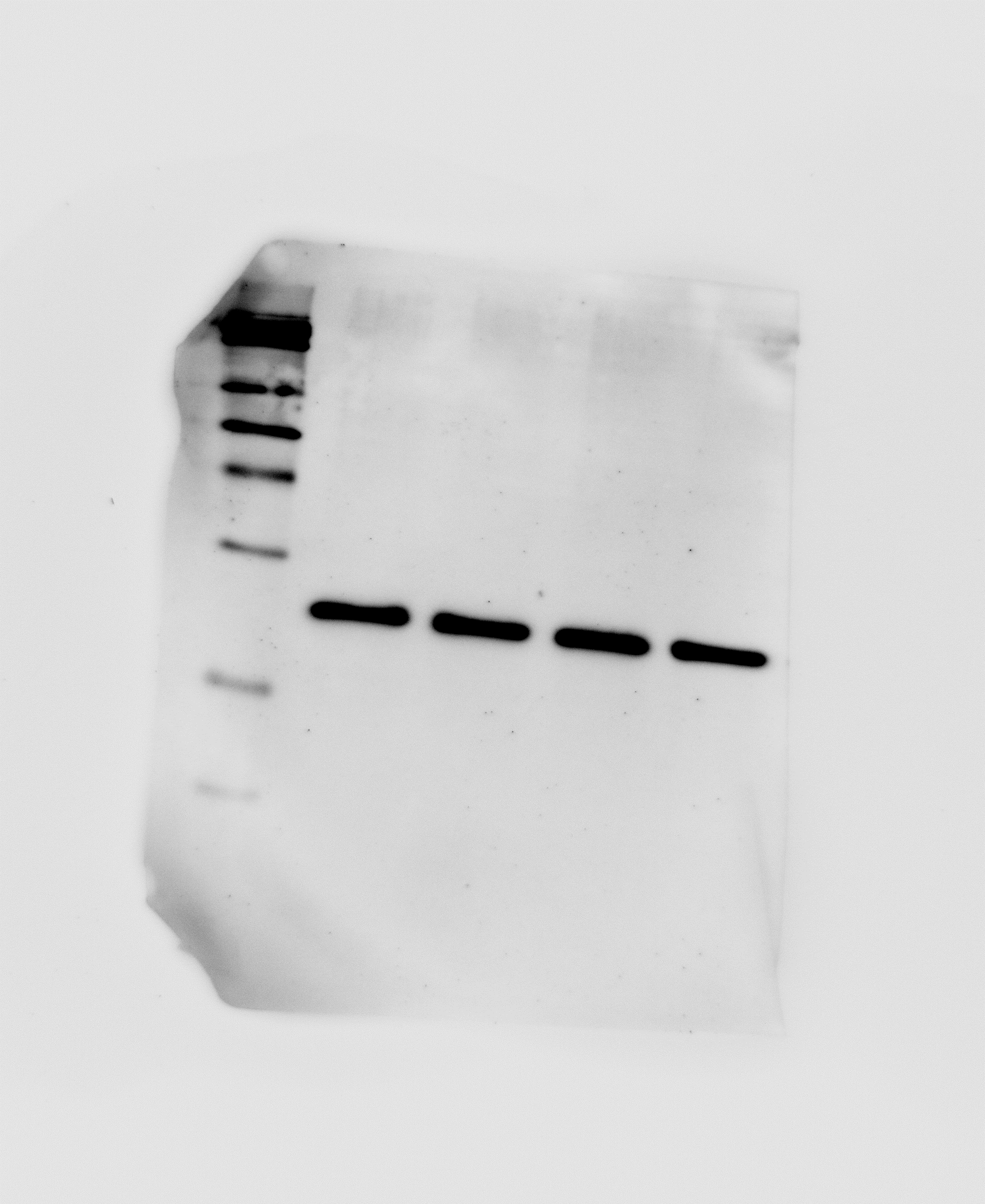

Supplement: Supplementary file 4 [file Supplementaryfile4.zip › Fig 5B/Saos-2/GAPDH-1.tif]

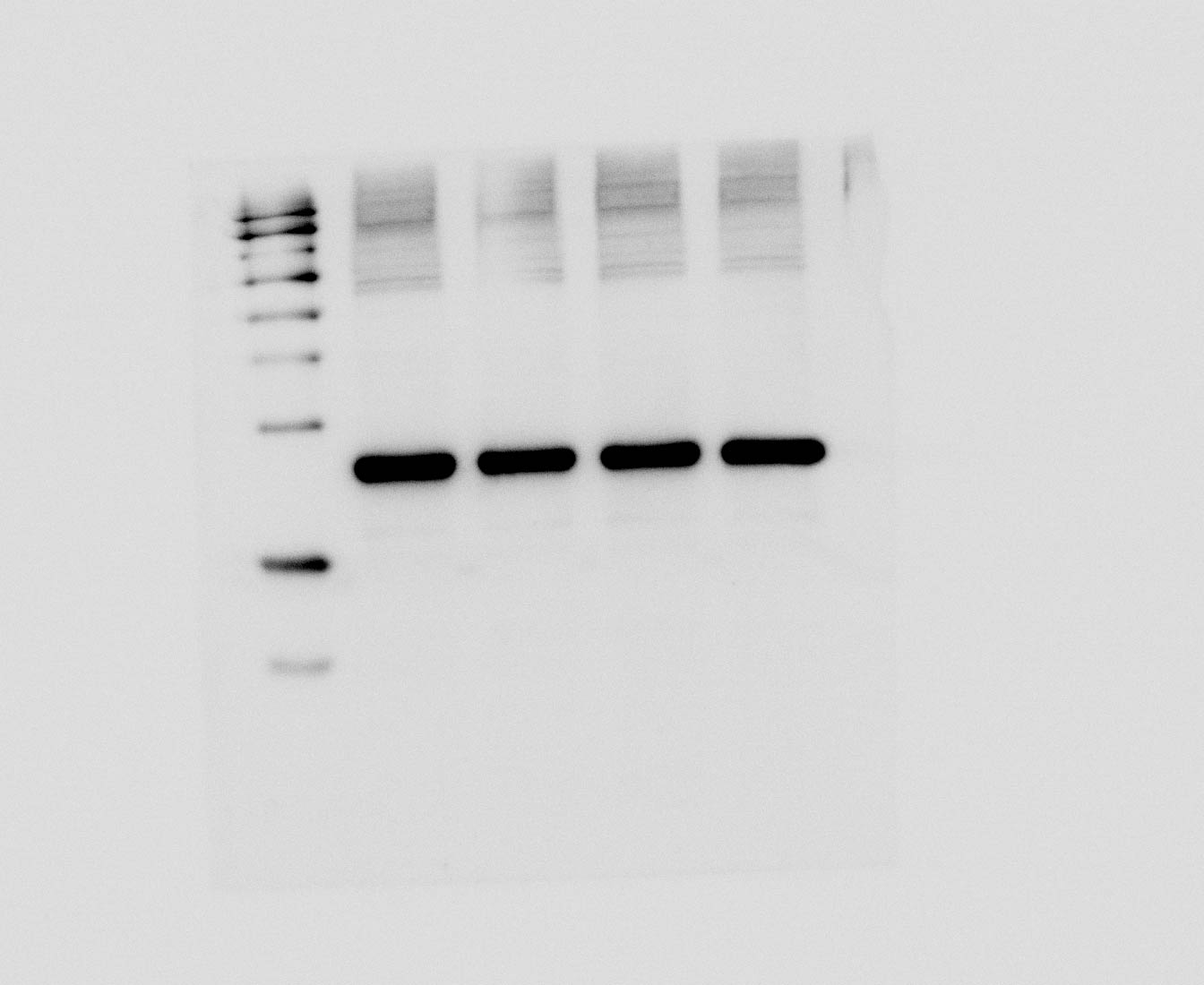

Supplement: Supplementary file 4 [file Supplementaryfile4.zip › Fig 5B/Saos-2/GAPDH-2.jpg]

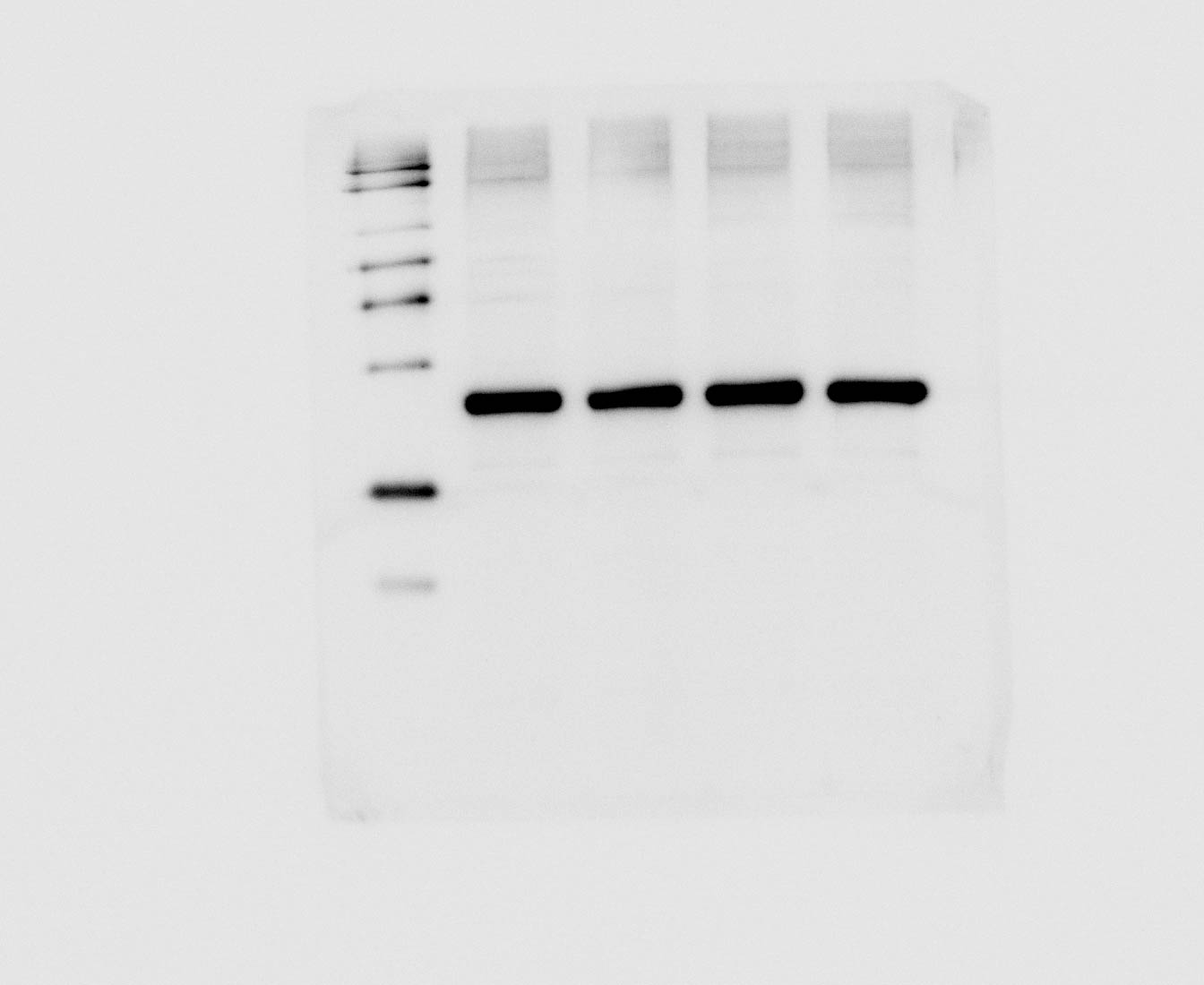

Supplement: Supplementary file 4 [file Supplementaryfile4.zip › Fig 5B/Saos-2/GAPDH-3 Report.jpg]

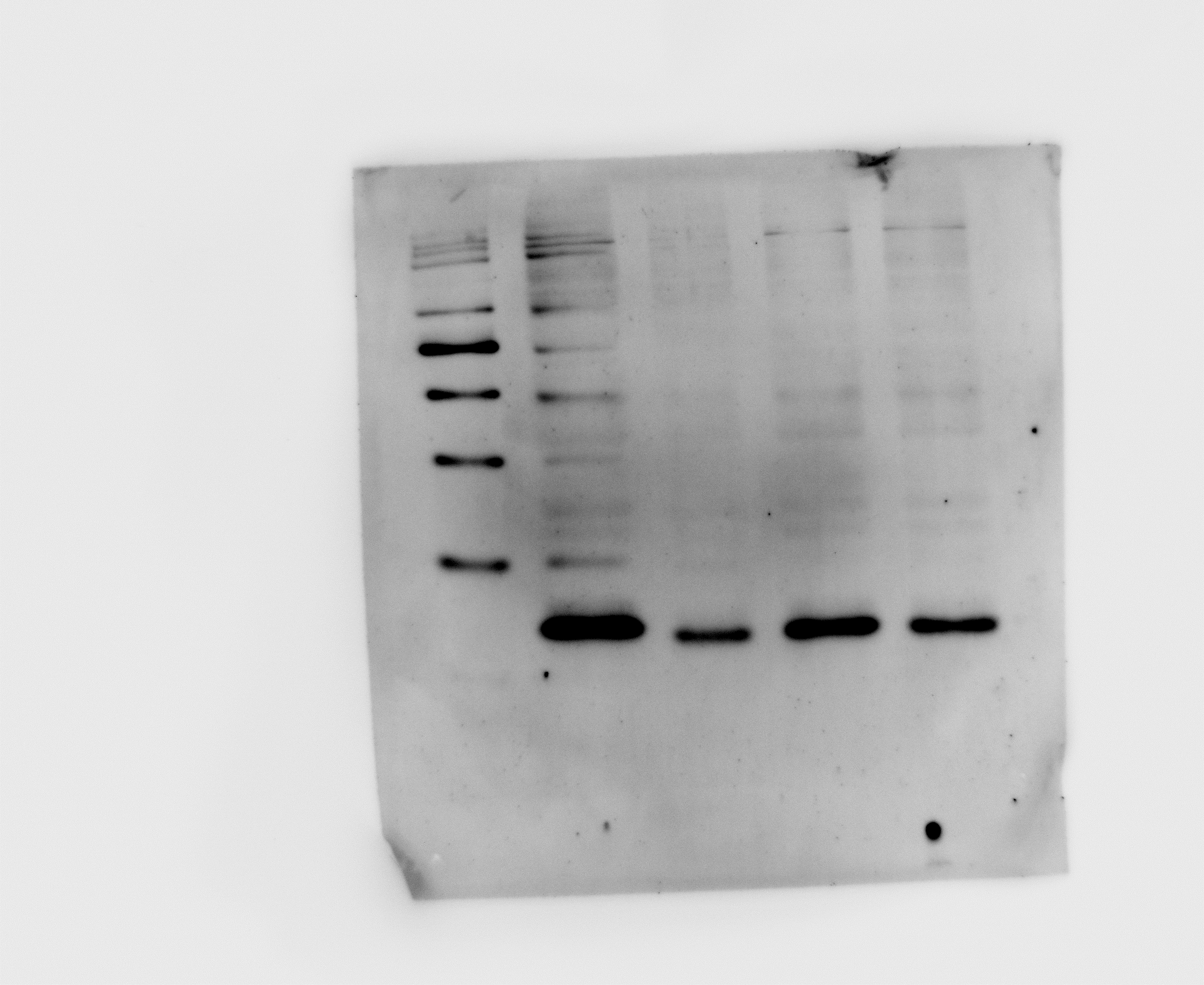

Supplement: Supplementary file 4 [file Supplementaryfile4.zip › Fig 5B/Saos-2/LCN2-1 Report.tif]

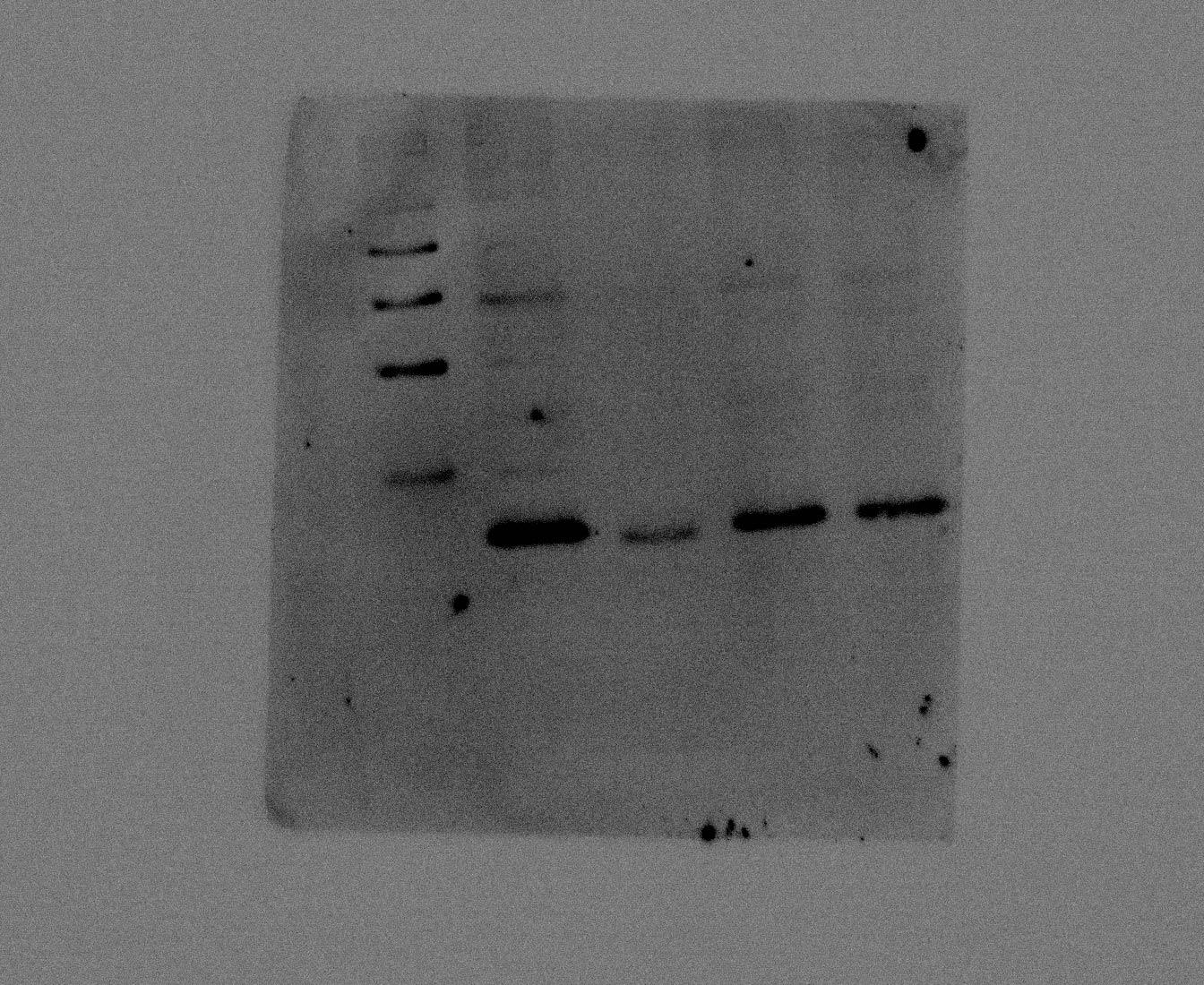

Supplement: Supplementary file 4 [file Supplementaryfile4.zip › Fig 5B/Saos-2/LCN2-2.jpg]

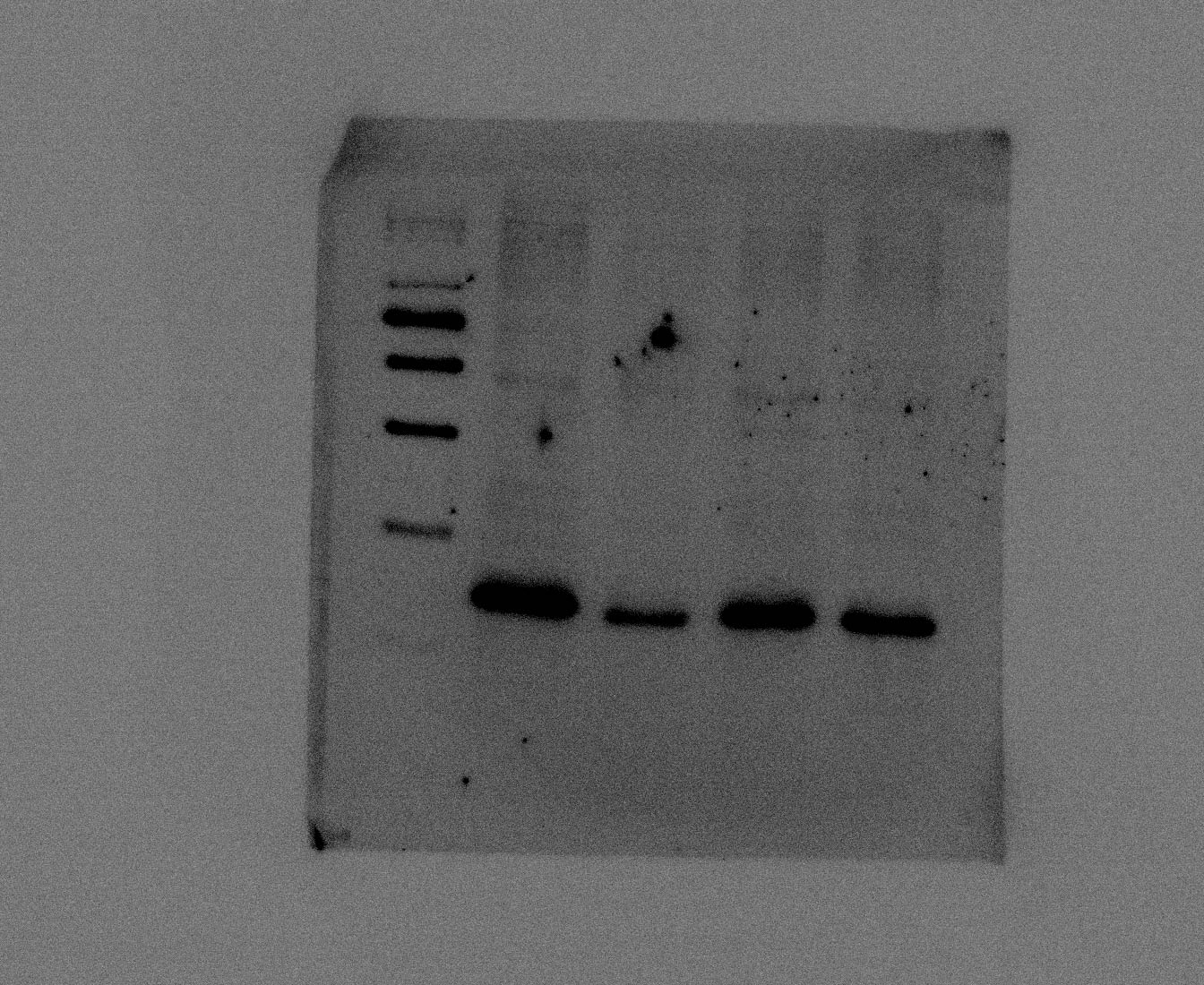

Supplement: Supplementary file 4 [file Supplementaryfile4.zip › Fig 5B/Saos-2/LCN2-3.jpg]

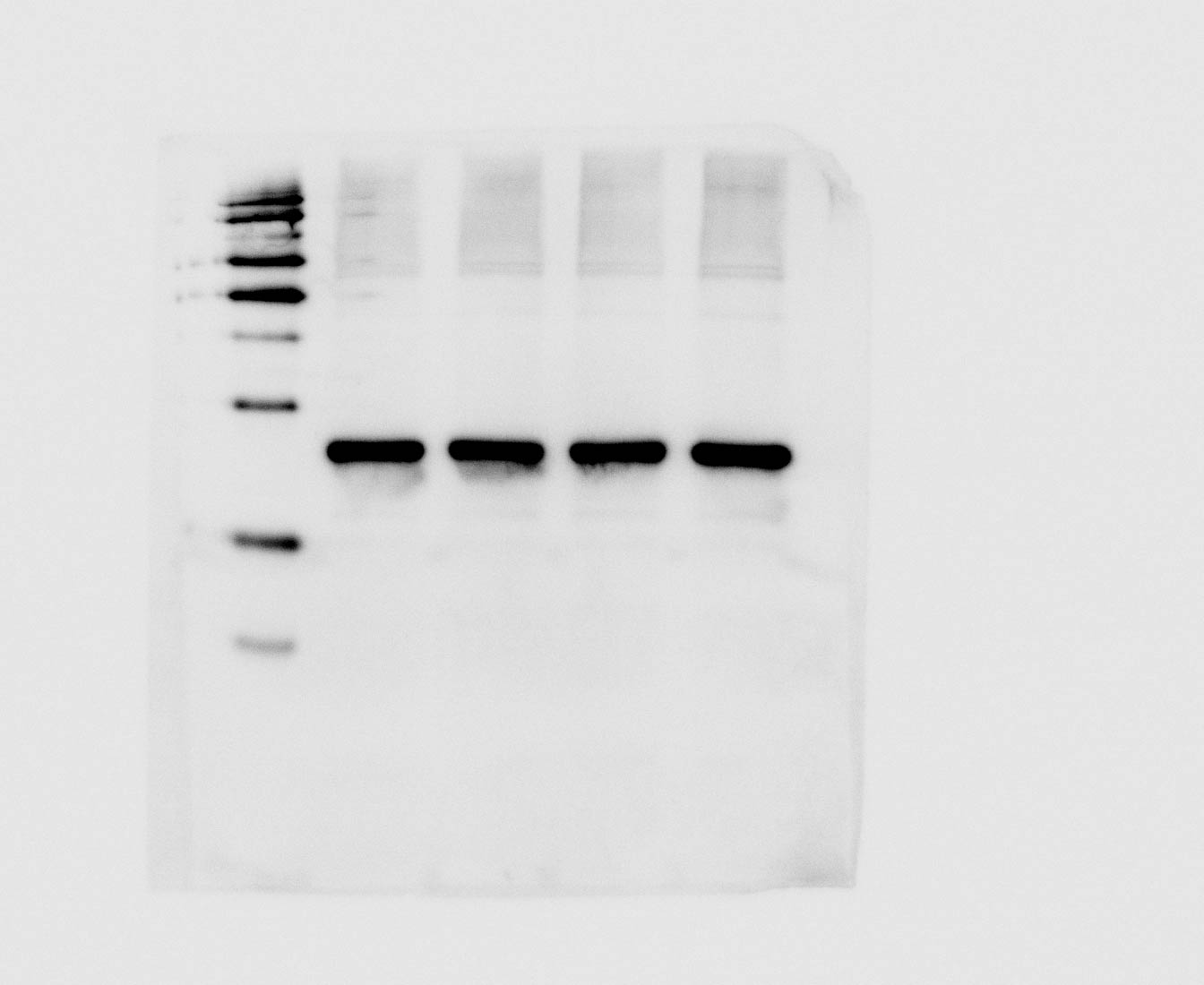

Supplement: Supplementary file 5 [file Supplementaryfile5.zip › Fig 6H/GAPDH-1 Report.jpg]

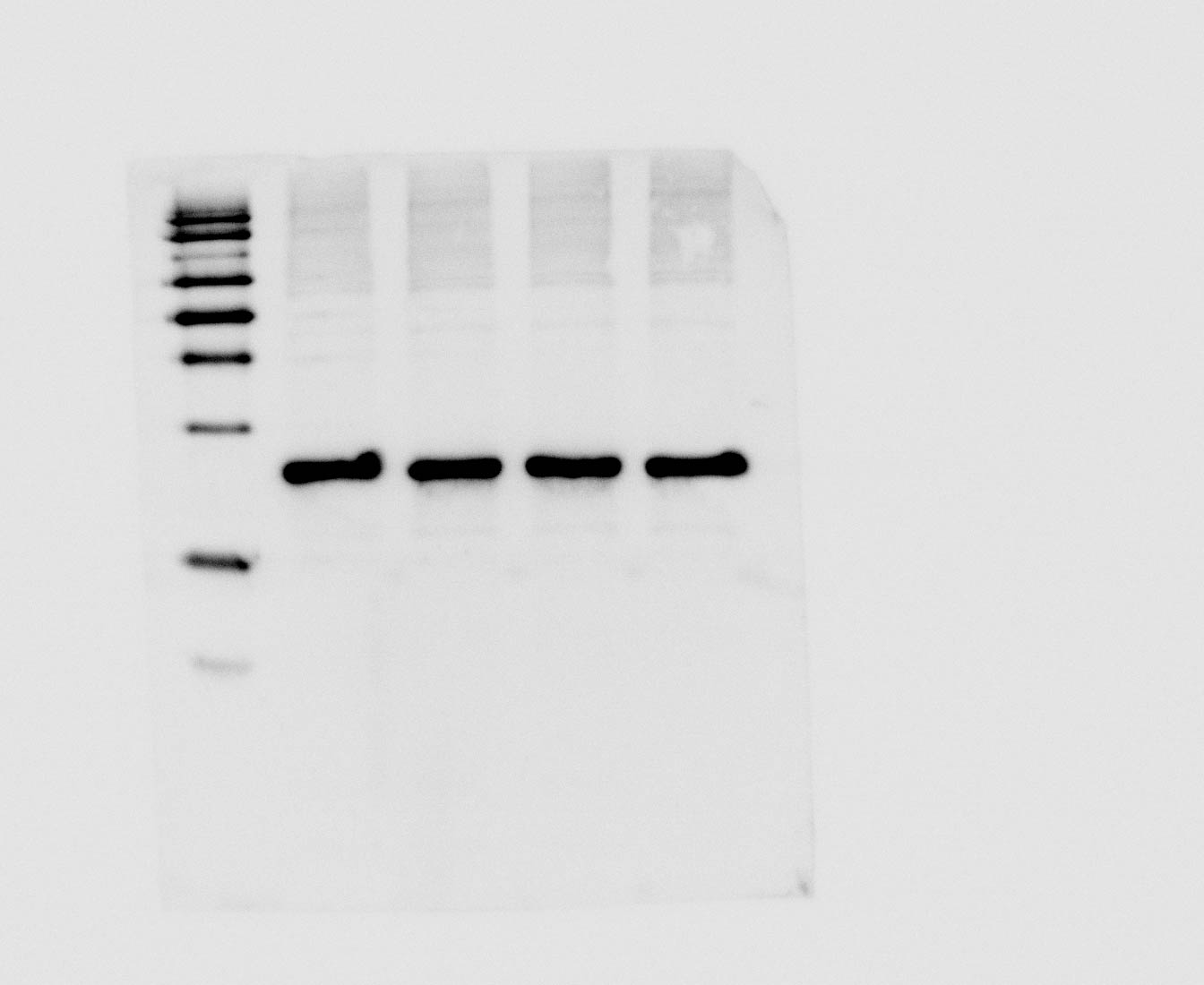

Supplement: Supplementary file 5 [file Supplementaryfile5.zip › Fig 6H/GAPDH-2.jpg]

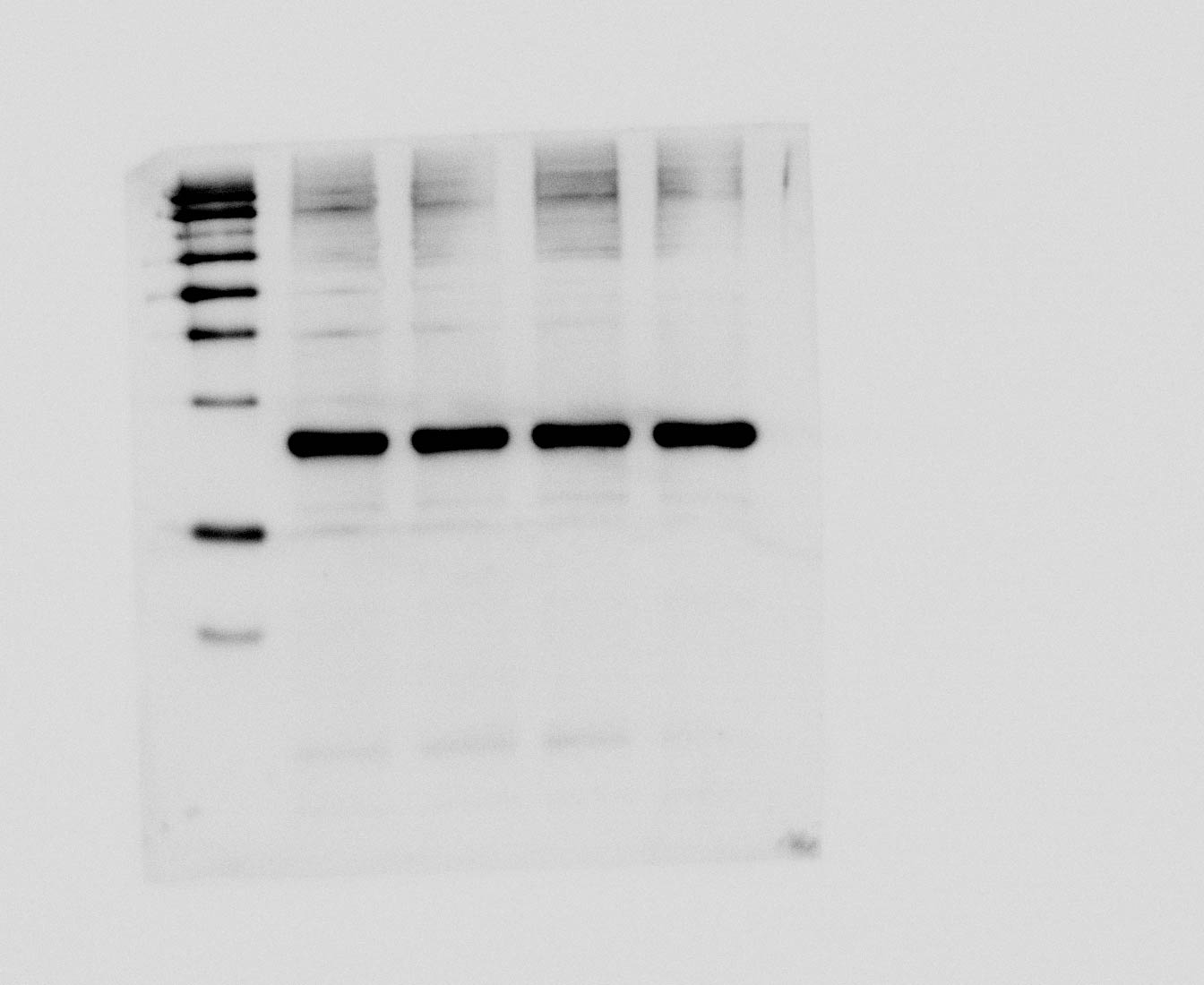

Supplement: Supplementary file 5 [file Supplementaryfile5.zip › Fig 6H/GAPDH-3.jpg]

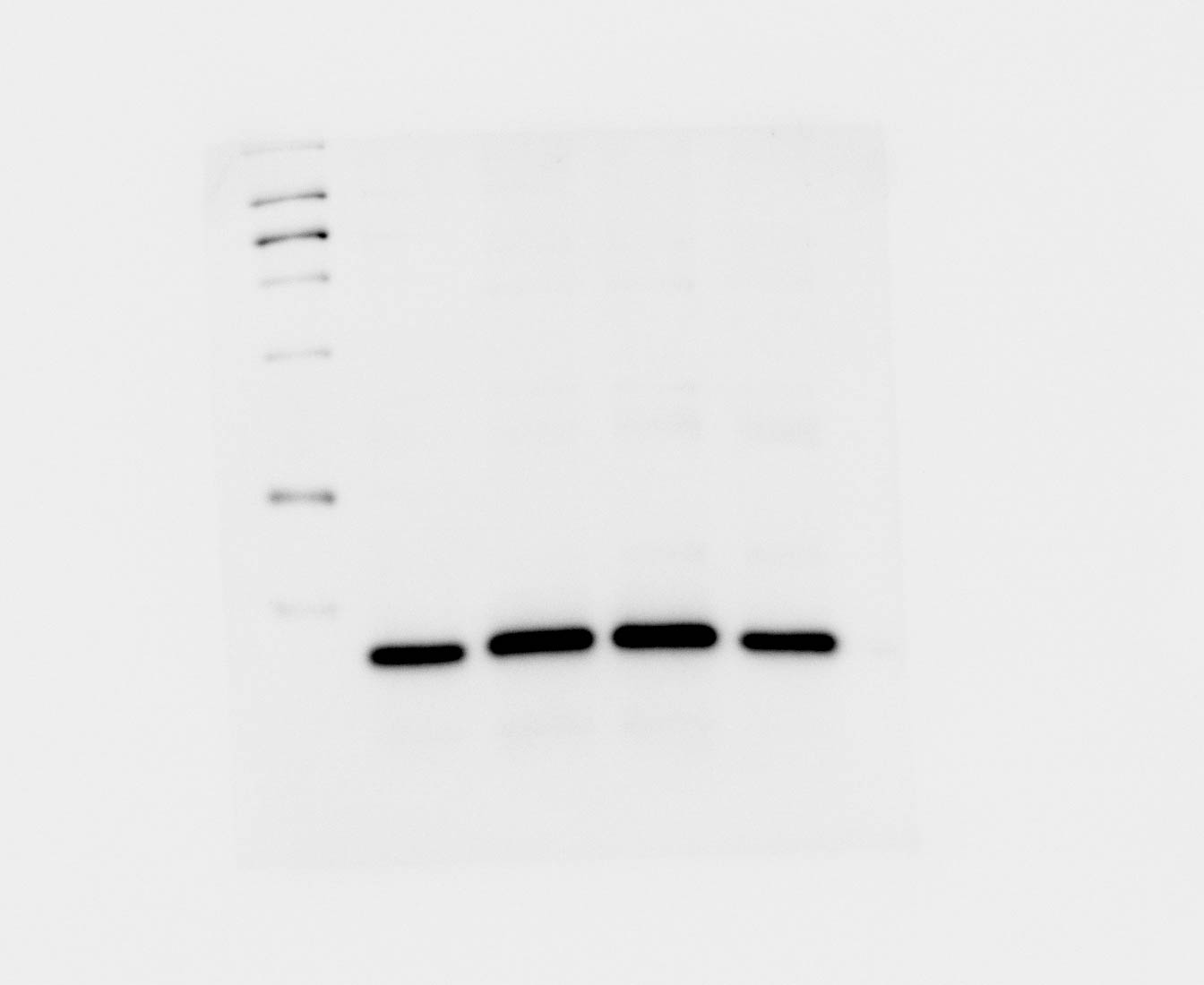

Supplement: Supplementary file 5 [file Supplementaryfile5.zip › Fig 6H/GPX4-1 Report.jpg]

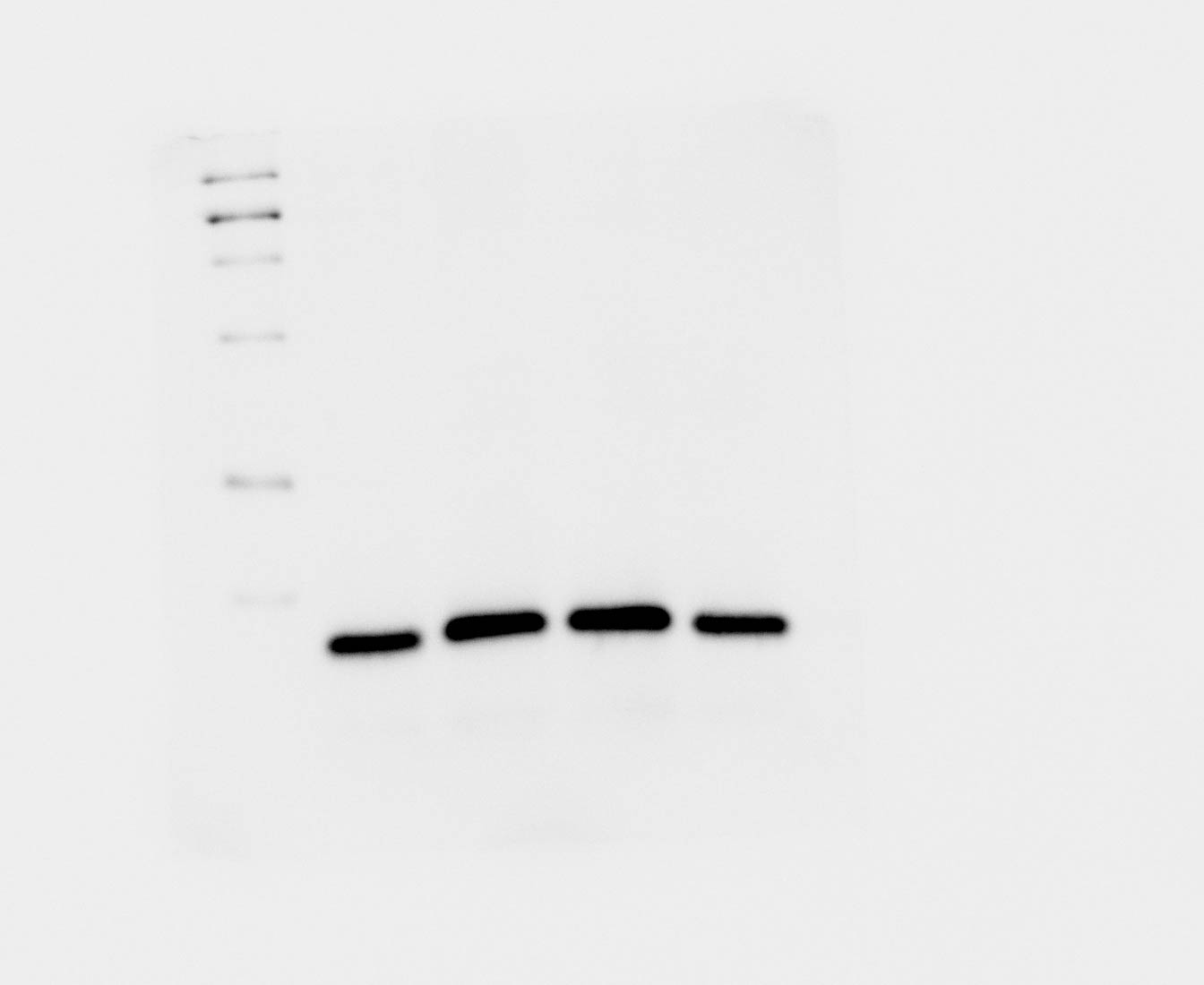

Supplement: Supplementary file 5 [file Supplementaryfile5.zip › Fig 6H/GPX4-2.jpg]

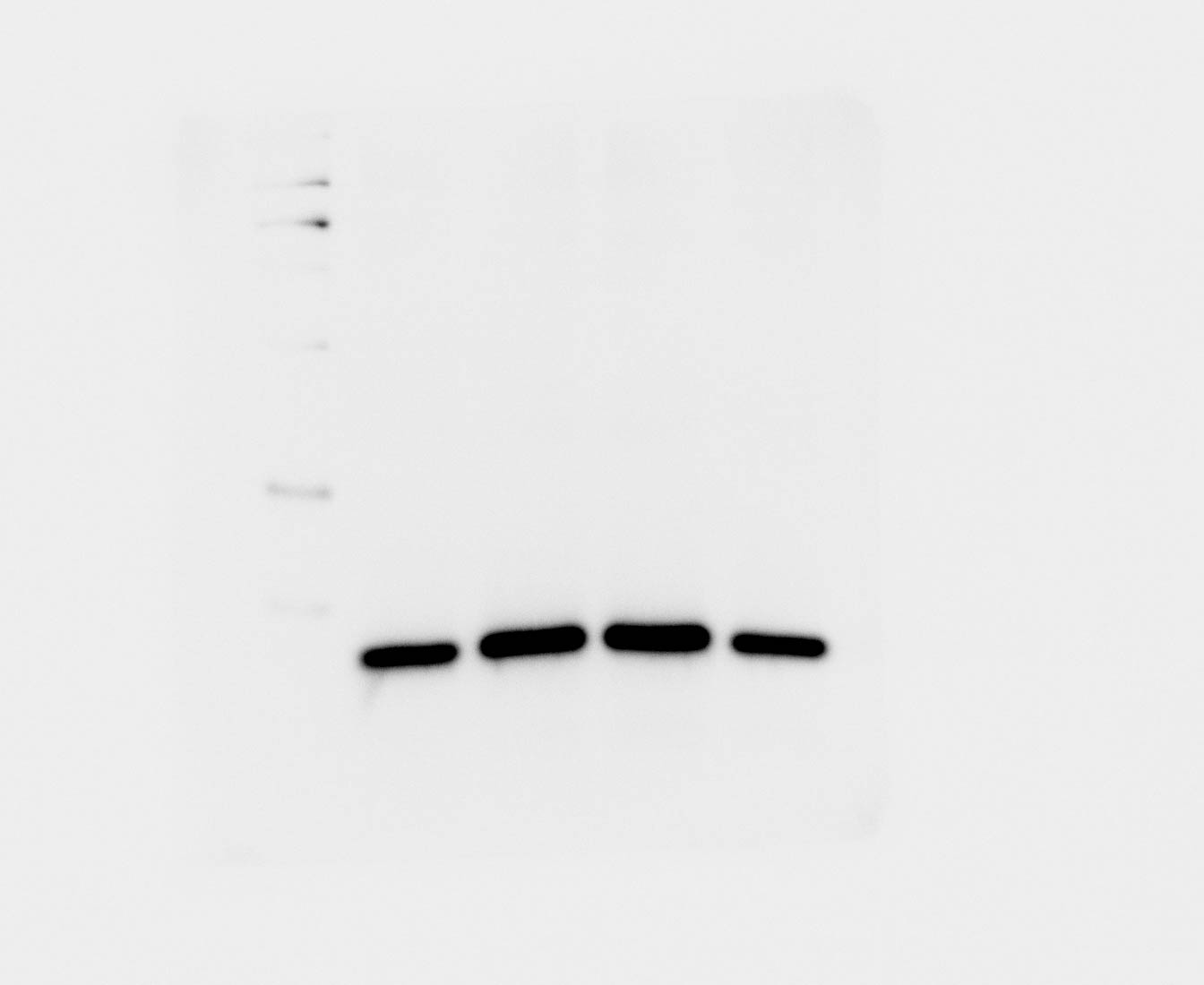

Supplement: Supplementary file 5 [file Supplementaryfile5.zip › Fig 6H/GPX4-3.jpg]

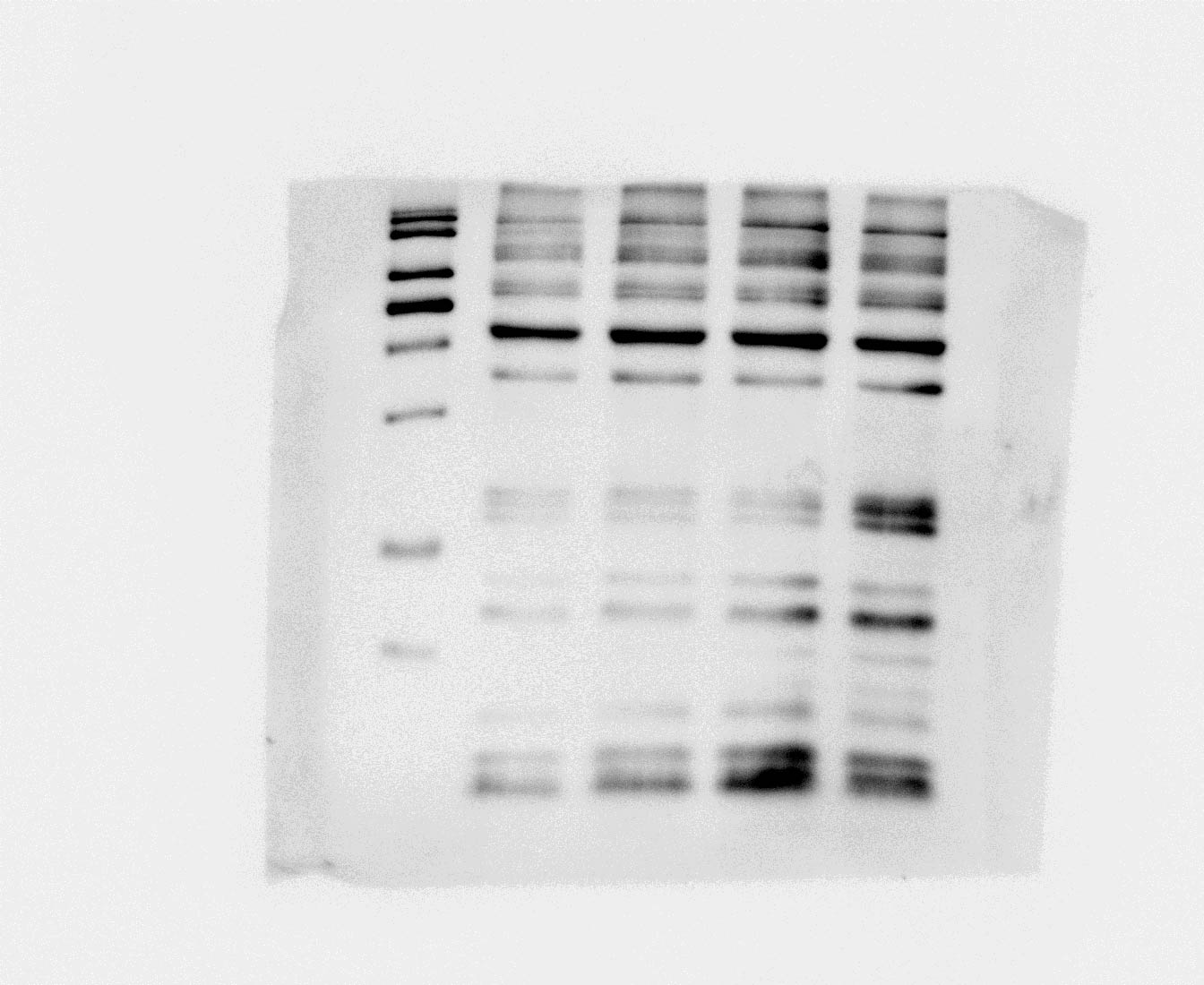

Supplement: Supplementary file 5 [file Supplementaryfile5.zip › Fig 6H/SLC7A11-1..jpg]

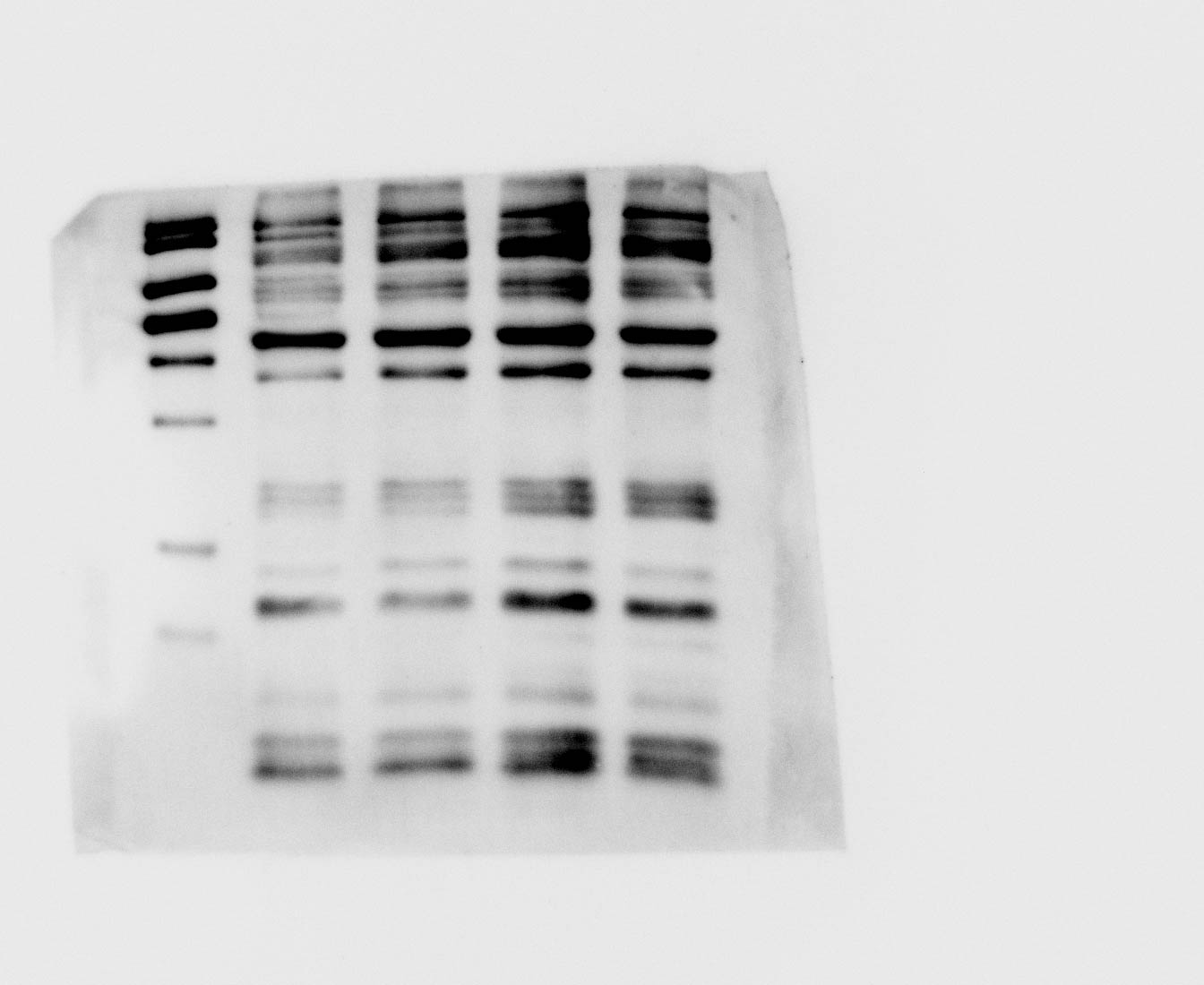

Supplement: Supplementary file 5 [file Supplementaryfile5.zip › Fig 6H/SLC7A11-2.jpg]

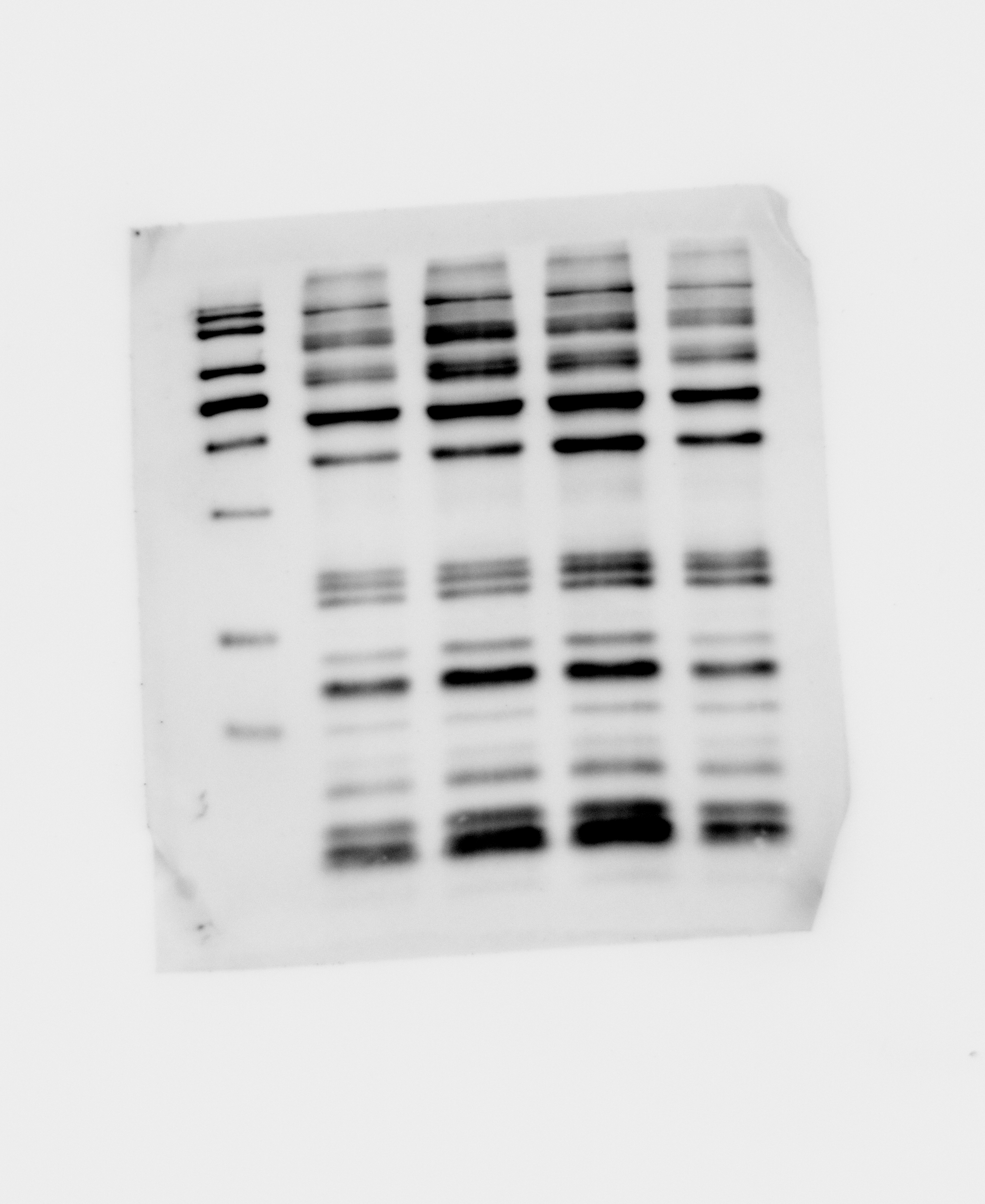

Supplement: Supplementary file 5 [file Supplementaryfile5.zip › Fig 6H/SLC7A11-3 Report.tif]

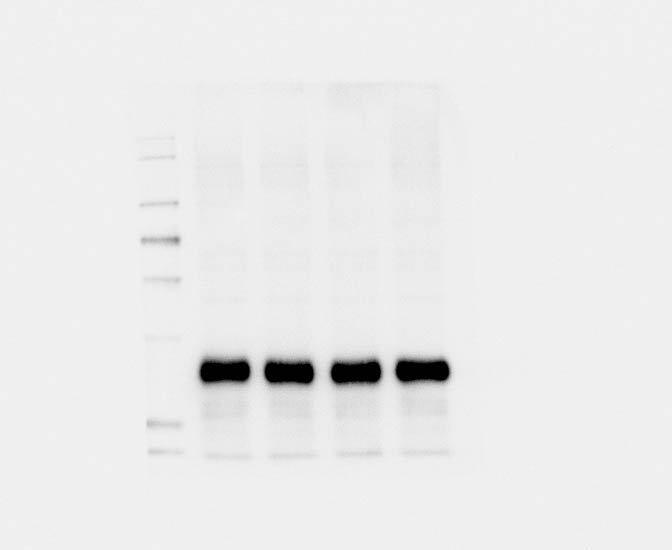

Supplement: Supplementary file 5 [file Supplementaryfile5.zip › Fig 6E/MG-63/GAPDH-1 Report.jpg]

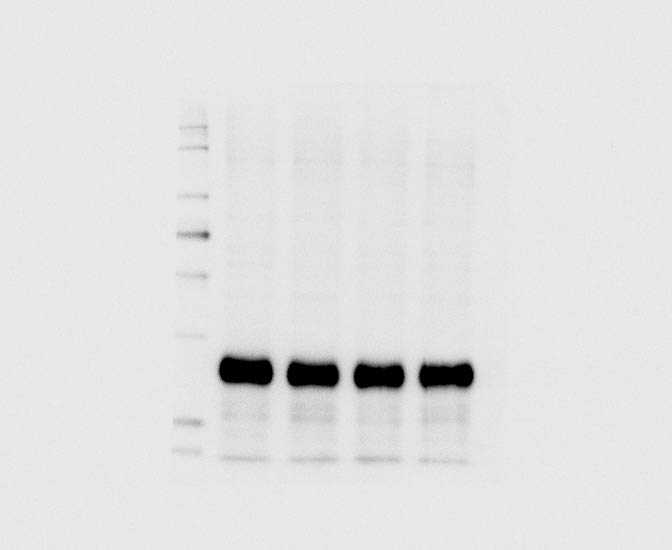

Supplement: Supplementary file 5 [file Supplementaryfile5.zip › Fig 6E/MG-63/GAPDH-2.jpg]

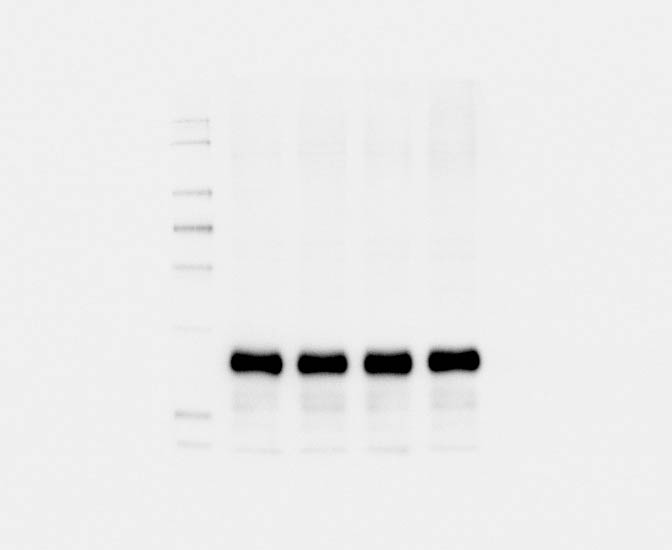

Supplement: Supplementary file 5 [file Supplementaryfile5.zip › Fig 6E/MG-63/GAPDH-3.jpg]

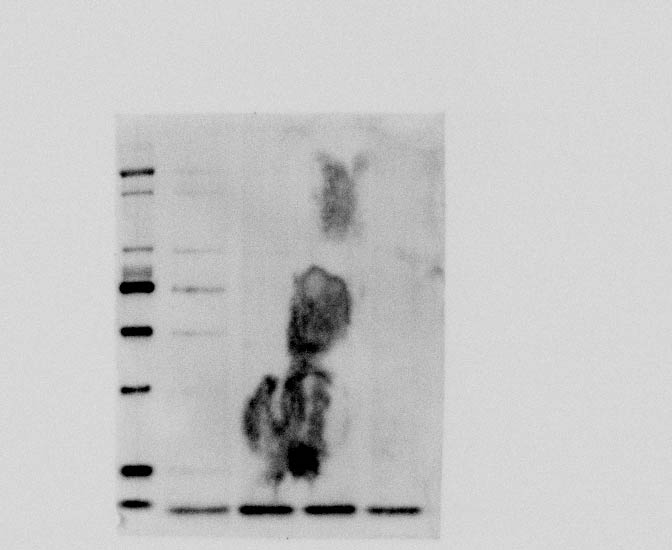

Supplement: Supplementary file 5 [file Supplementaryfile5.zip › Fig 6E/MG-63/GPX4-1.jpg]

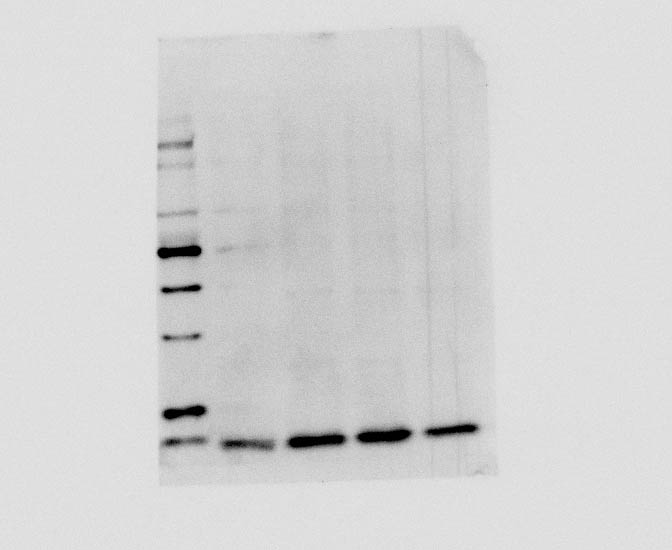

Supplement: Supplementary file 5 [file Supplementaryfile5.zip › Fig 6E/MG-63/GPX4-2 Report.jpg]

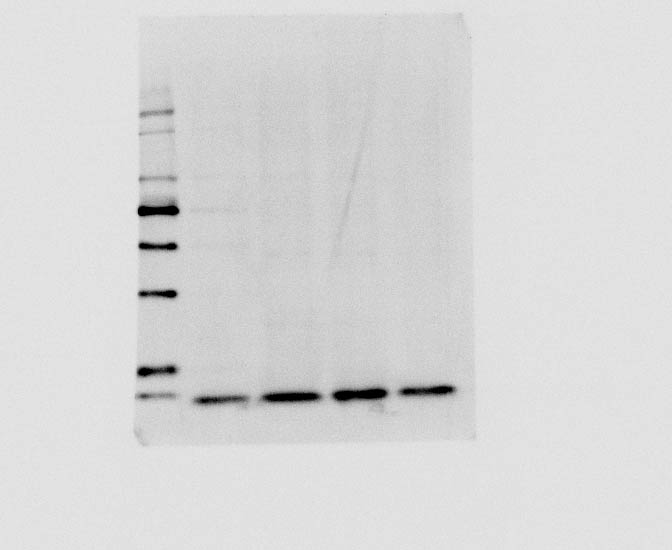

Supplement: Supplementary file 5 [file Supplementaryfile5.zip › Fig 6E/MG-63/GPX4-3.jpg]

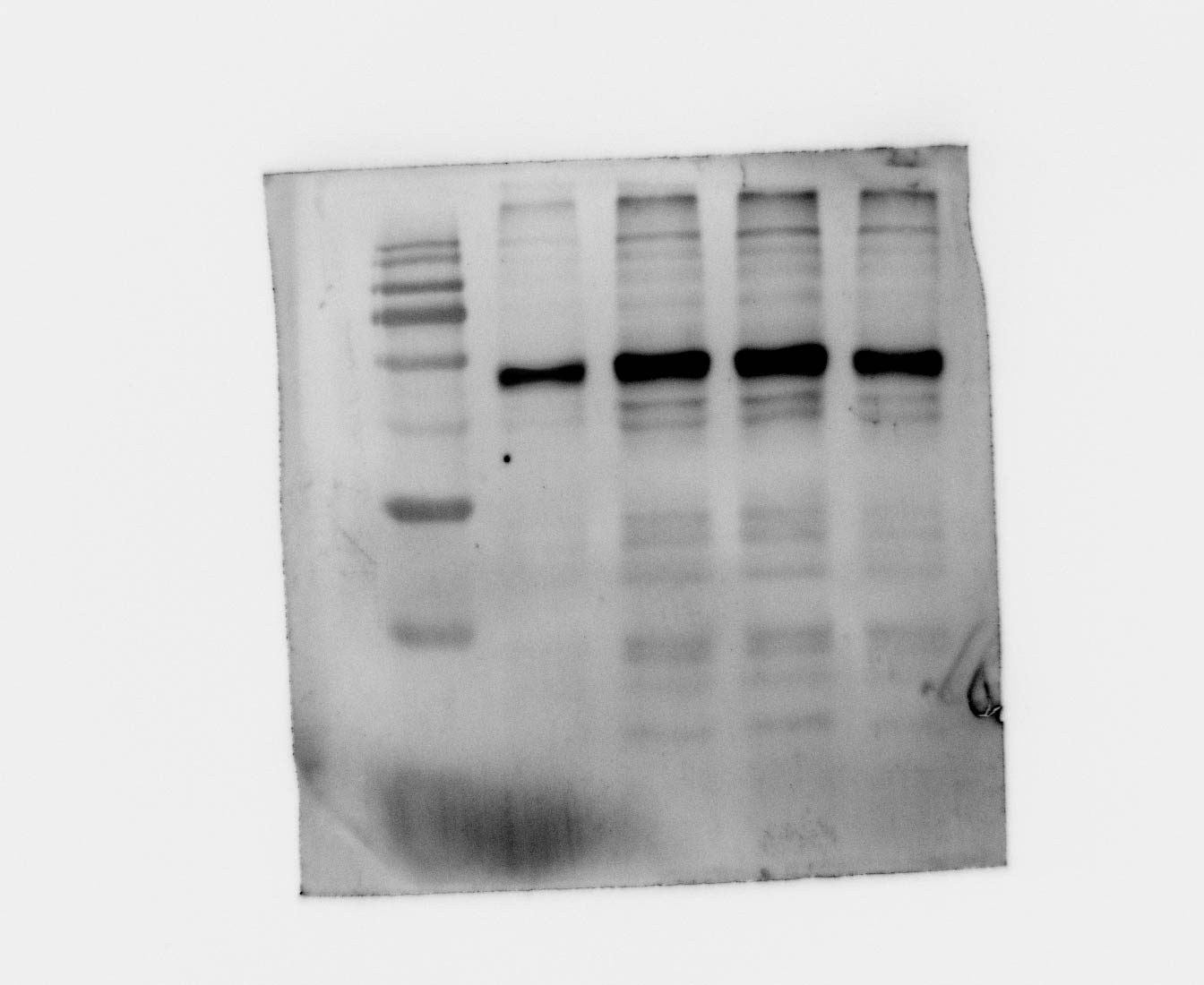

Supplement: Supplementary file 5 [file Supplementaryfile5.zip › Fig 6E/MG-63/SLC7A11-1.jpg]

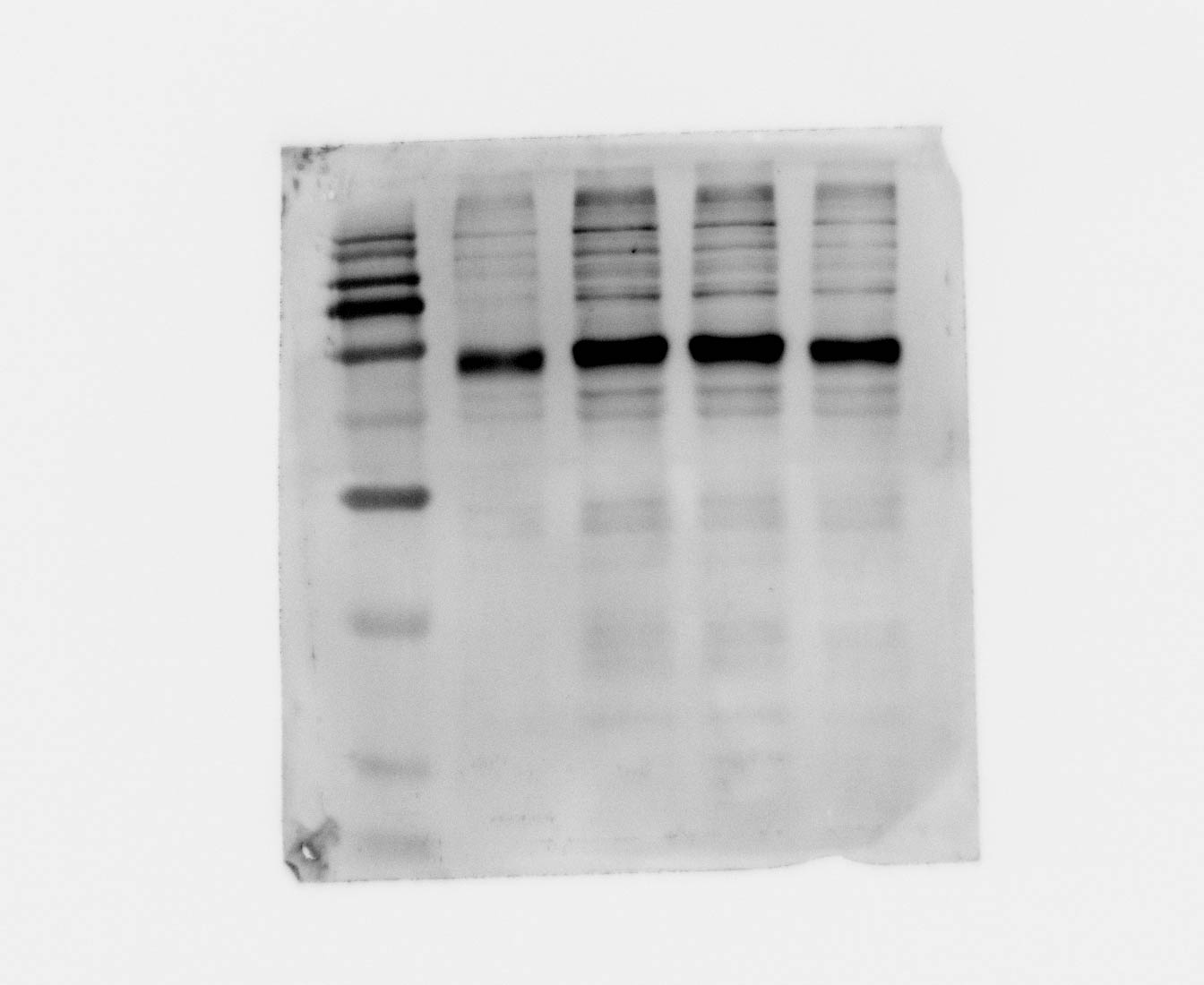

Supplement: Supplementary file 5 [file Supplementaryfile5.zip › Fig 6E/MG-63/SLC7A11-2.jpg]

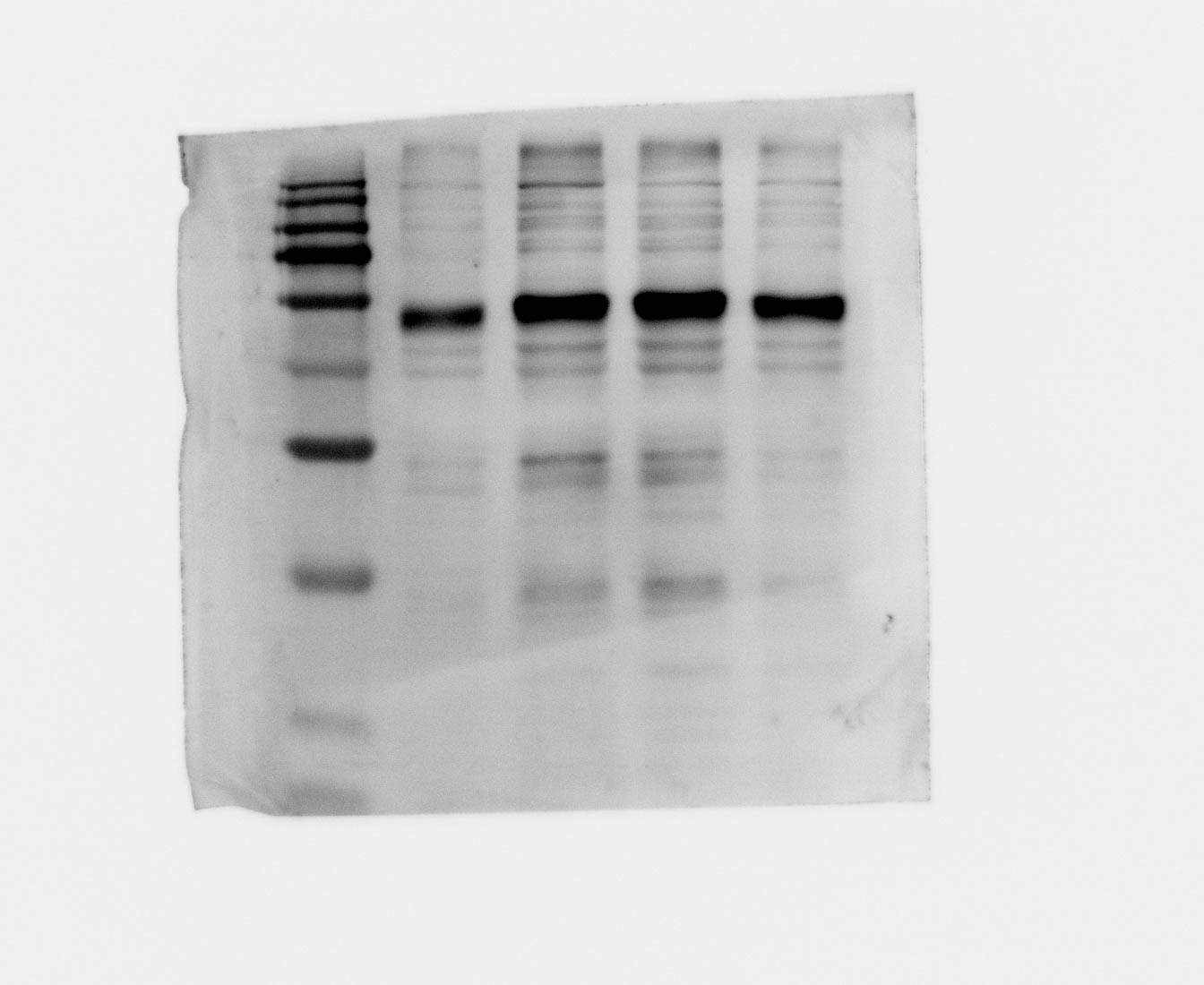

Supplement: Supplementary file 5 [file Supplementaryfile5.zip › Fig 6E/MG-63/SLC7A11-3 Report.jpg]

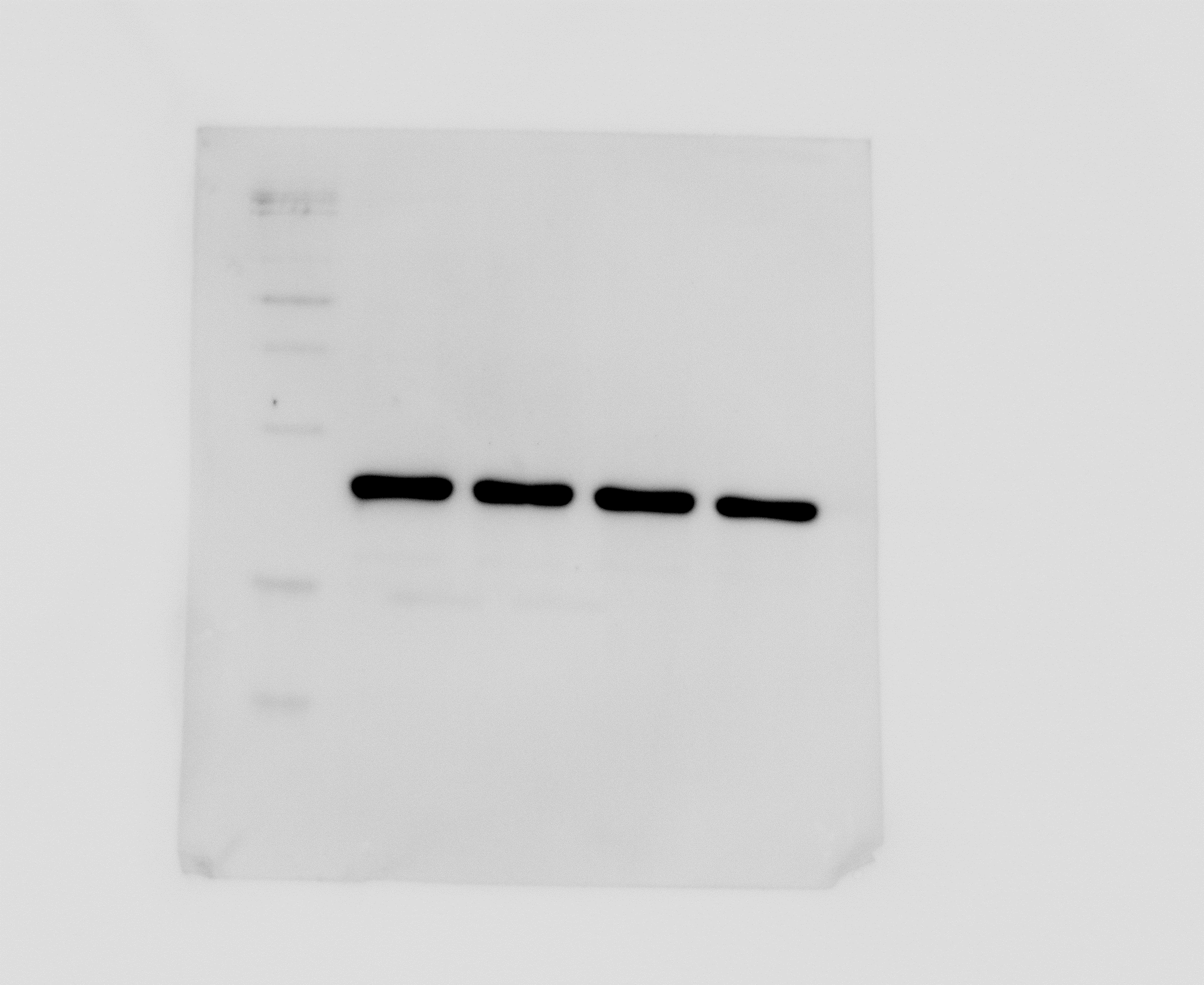

Supplement: Supplementary file 5 [file Supplementaryfile5.zip › Fig 6E/Saos-2/GAPDH-1.tif]

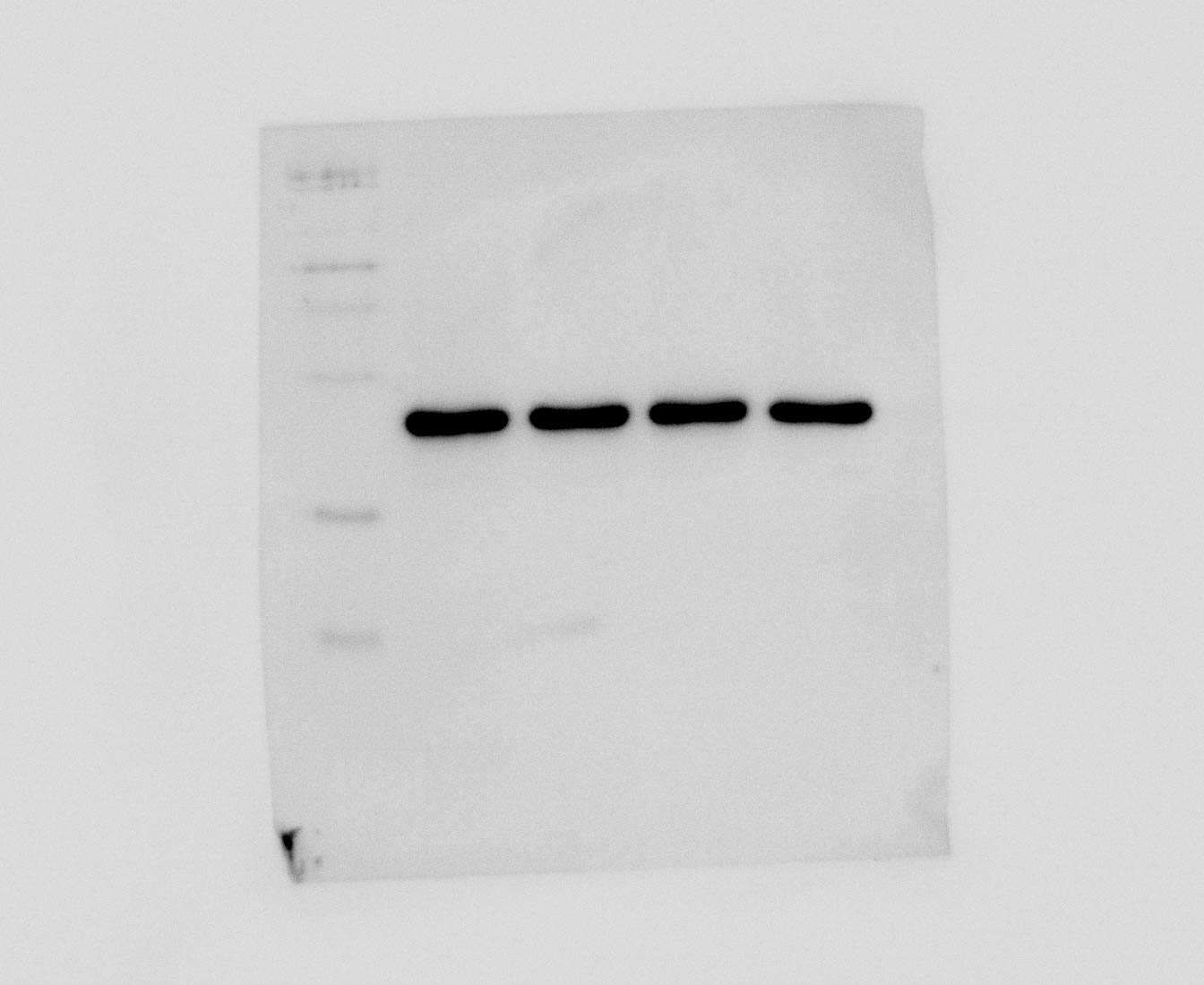

Supplement: Supplementary file 5 [file Supplementaryfile5.zip › Fig 6E/Saos-2/GAPDH-2.jpg]

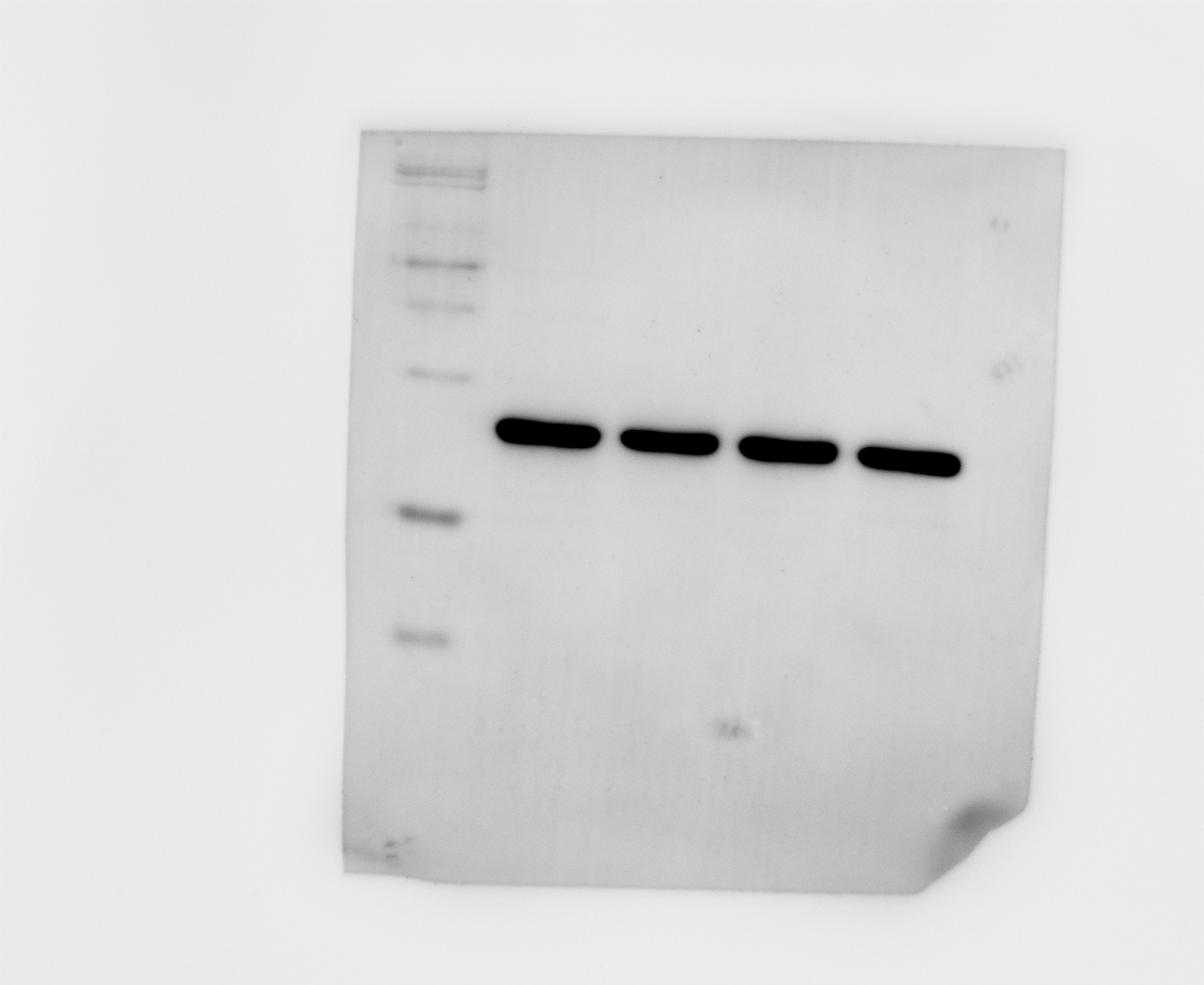

Supplement: Supplementary file 5 [file Supplementaryfile5.zip › Fig 6E/Saos-2/GAPDH-3 Report.tif]

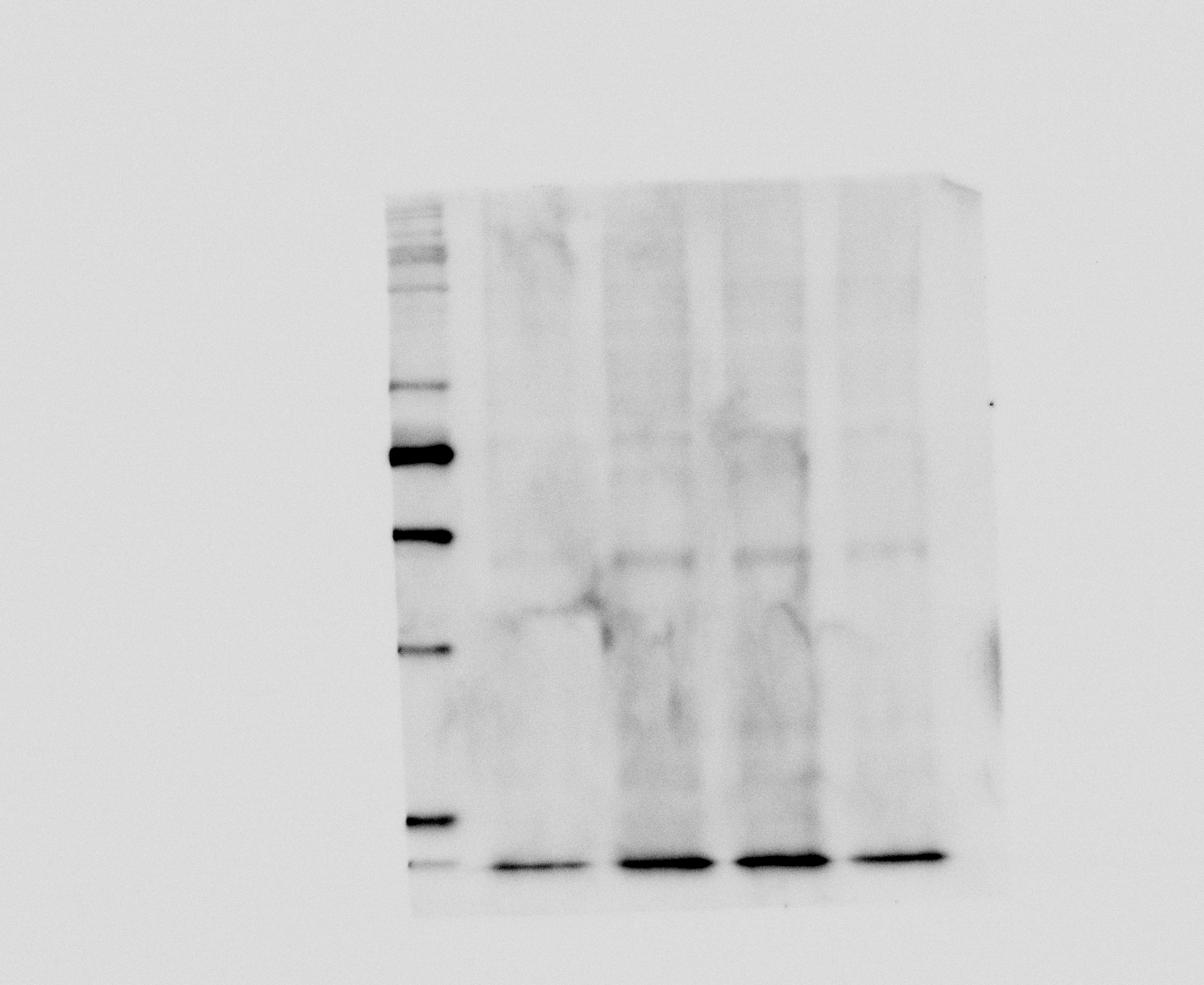

Supplement: Supplementary file 5 [file Supplementaryfile5.zip › Fig 6E/Saos-2/GPX4-1 Report.tif]

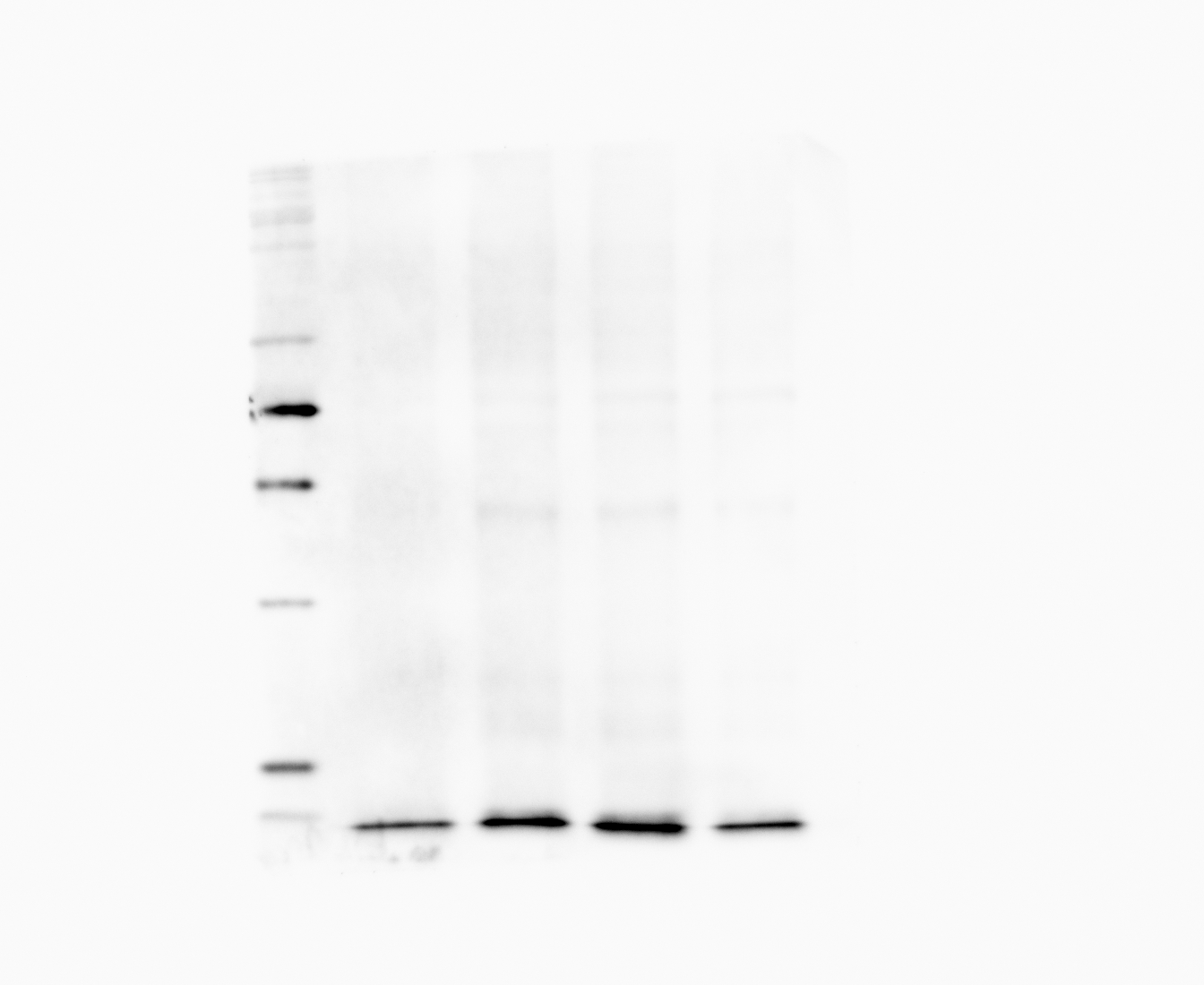

Supplement: Supplementary file 5 [file Supplementaryfile5.zip › Fig 6E/Saos-2/GPX4-2.tif]

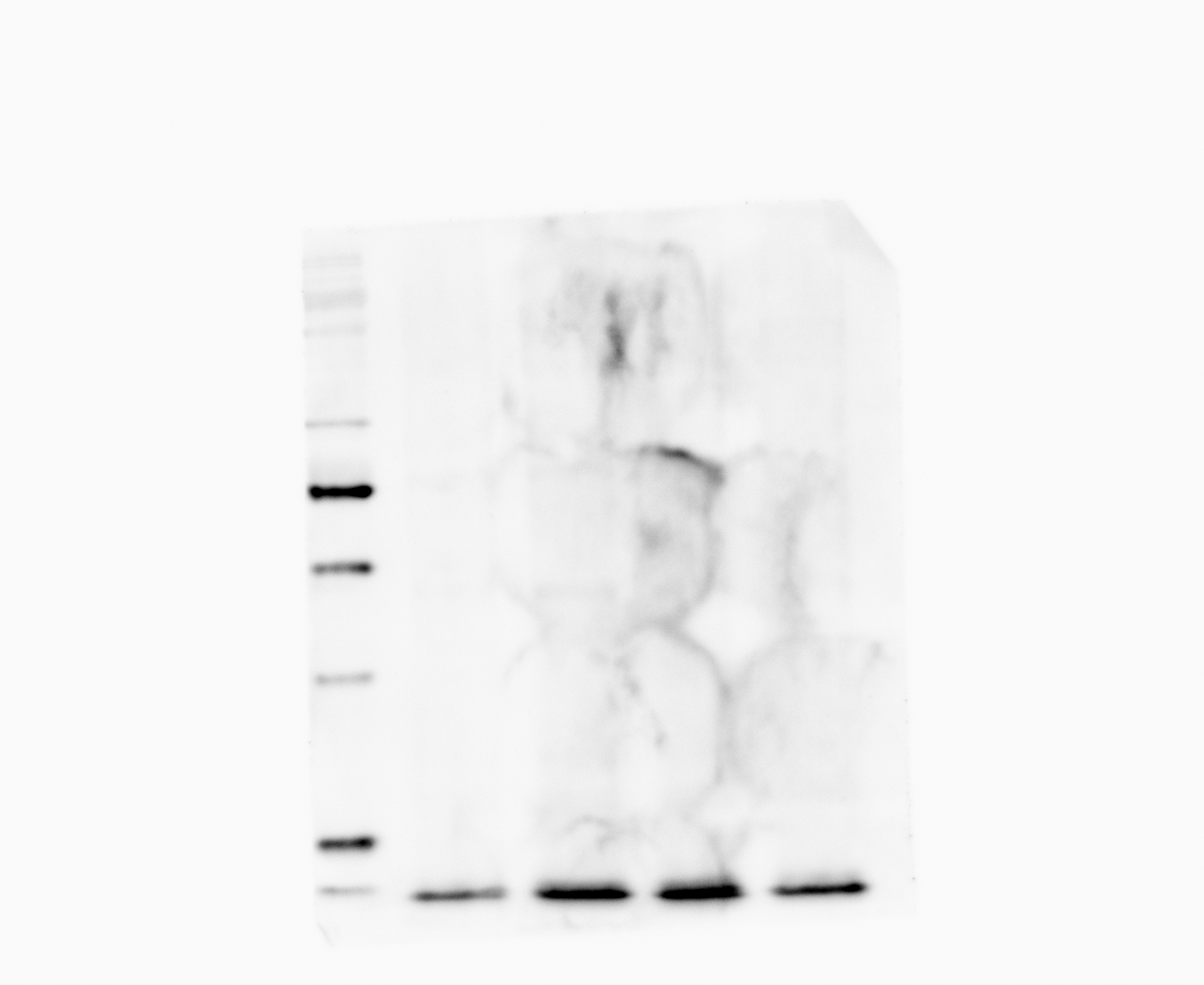

Supplement: Supplementary file 5 [file Supplementaryfile5.zip › Fig 6E/Saos-2/GPX4-3.tif]

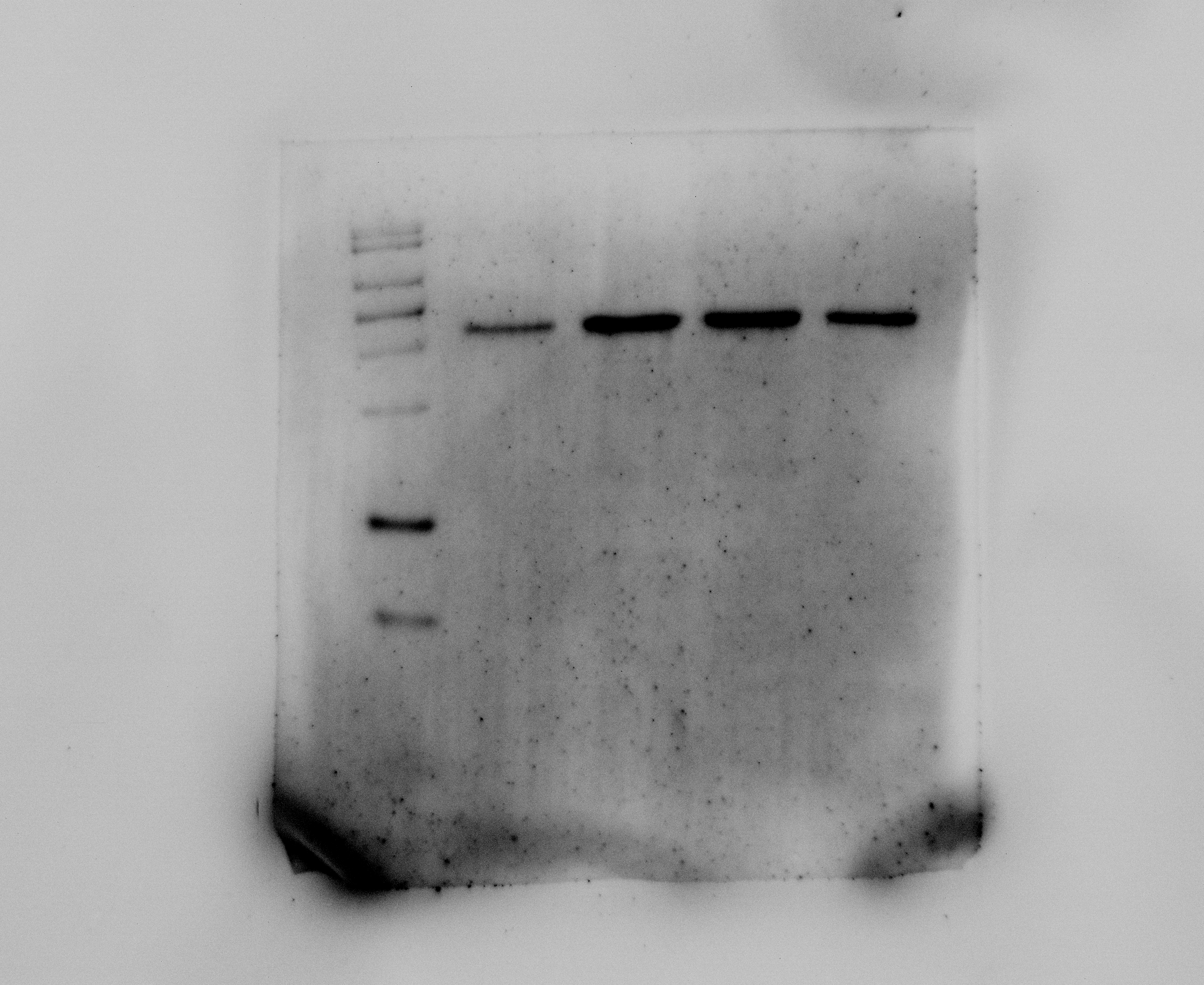

Supplement: Supplementary file 5 [file Supplementaryfile5.zip › Fig 6E/Saos-2/SLC7A11-1..tif]

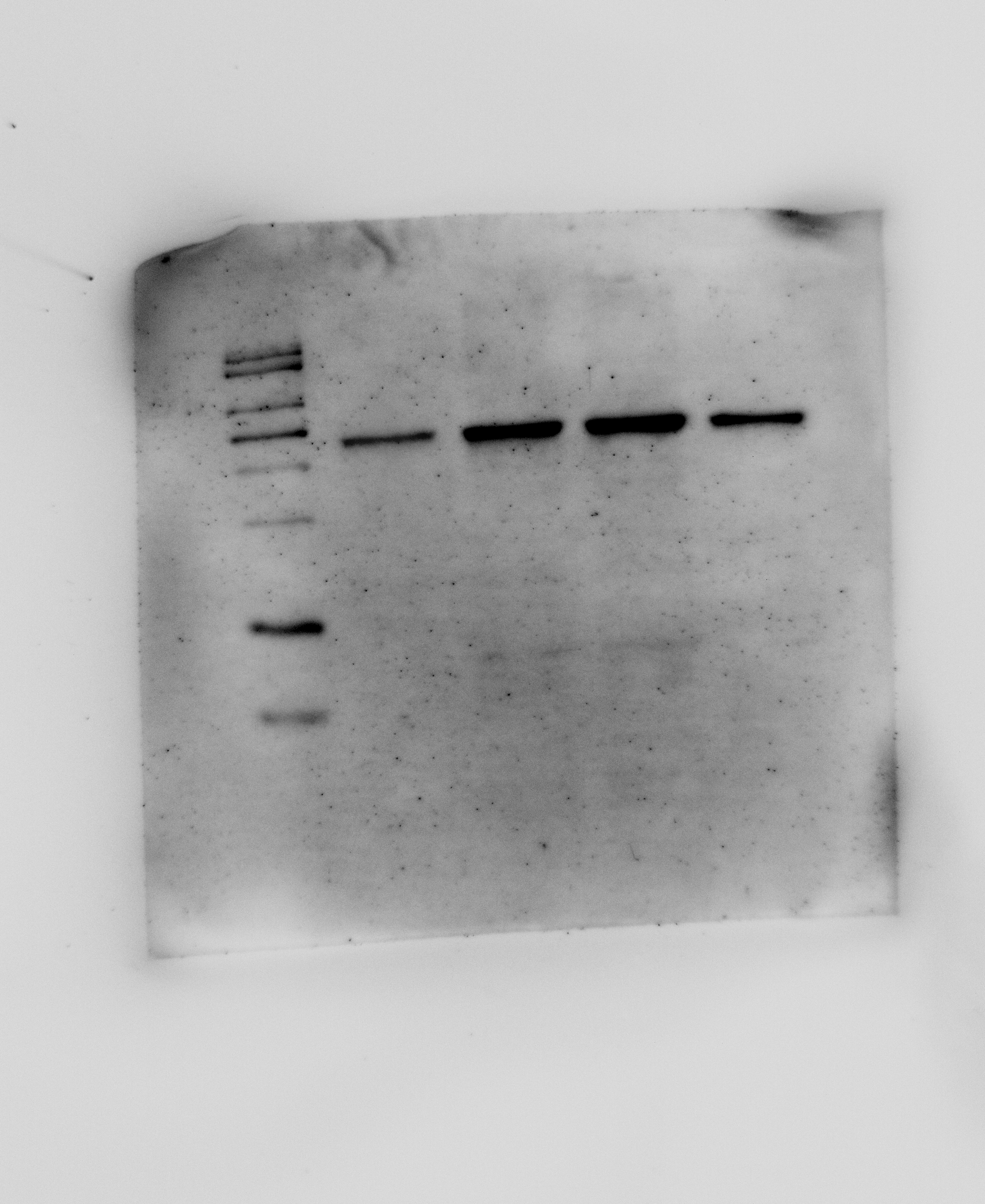

Supplement: Supplementary file 5 [file Supplementaryfile5.zip › Fig 6E/Saos-2/SLC7A11-2 Report.tif]

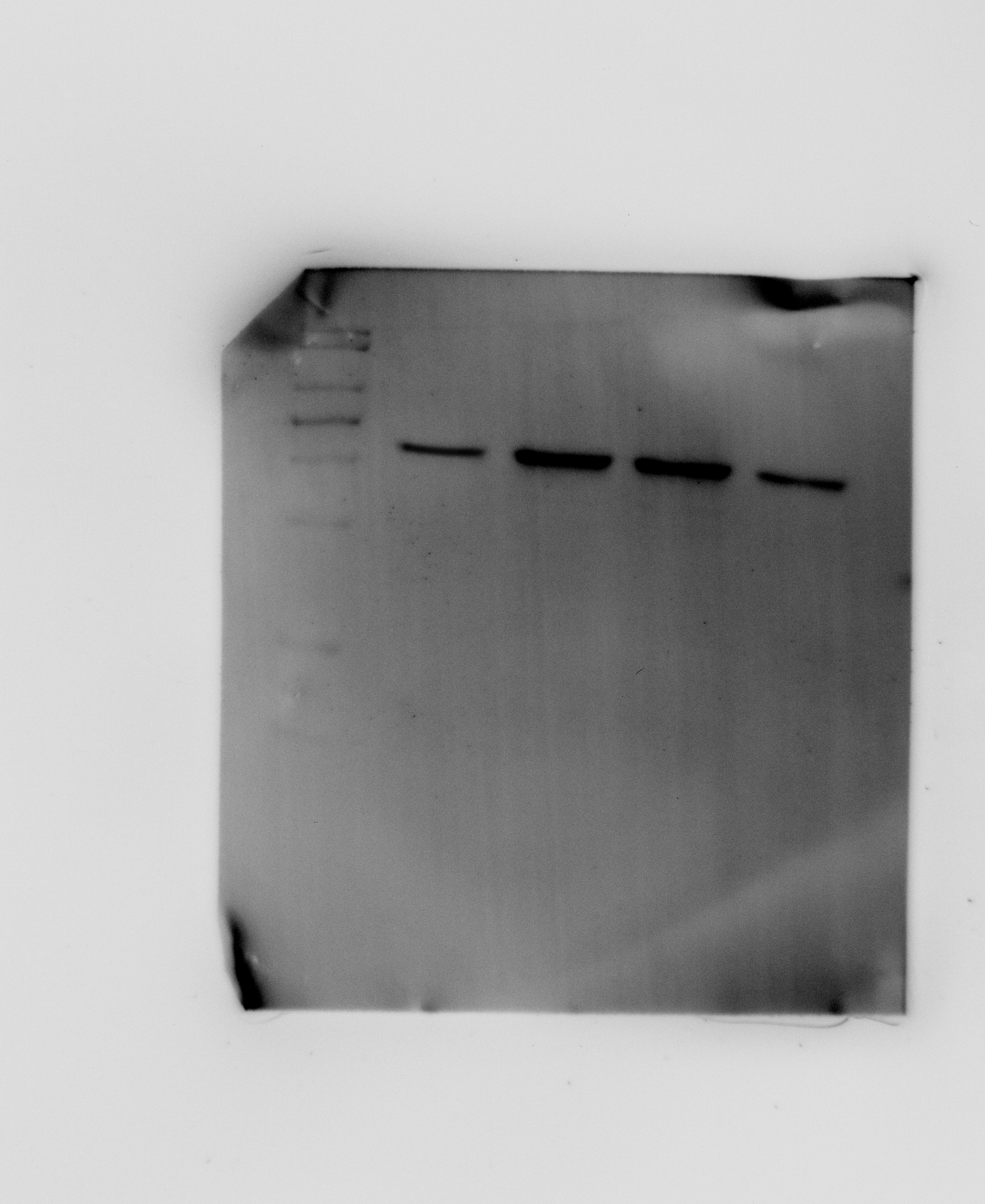

Supplement: Supplementary file 5 [file Supplementaryfile5.zip › Fig 6E/Saos-2/SLC7A11-3.tif]
